# Colour bio-factories: Towards scale-up production of anthocyanins in plant cell cultures

**DOI:** 10.1016/j.ymben.2018.06.004

**Published:** 2018-07

**Authors:** Ingo Appelhagen, Anders Keim Wulff-Vester, Micael Wendell, Anne-Kathrine Hvoslef-Eide, Julia Russell, Anne Oertel, Stefan Martens, Hans-Peter Mock, Cathie Martin, Andrea Matros

**Affiliations:** a John Innes Centre, Department of Metabolic Biology, Norwich Research Park, Norwich NR47UH, United Kingdom; b Norwegian University of Life Sciences, Faculty of Biosciences, Department of Plant Sciences, Fougnerbakken 3, N-1432 Ås, Norway; c Leibniz Institute of Plant Genetics and Crop Plant Research (IPK-Gatersleben), Department of Physiology and Cell Biology, Corrensstraße 3, 06466 Stadt Seeland, OT Gatersleben, Germany; d TransMIT GmbH, Project division PlantMetaChem, Kerkrader Str. 3, 35394 Giessen, Germany; e Edmund Mach Foundation, Research and Innovation Centre, Department of Food Quality and Nutrition, Via E. Mach, 1 38010 San Michele all'Adige, TN, Italy

**Keywords:** C3R, cyanidin 3-*O*-rutinoside, C3couR, cyanidin 3-*O*-(coumaroyl) rutinoside, C3ferR, cyanidin 3-*O*-(feruloyl) rutinoside, D3R, delphinidin 3-*O*-rutinoside, Pel3R, pelargonidin 3-*O*-rutinoside, Peo3R, peonidin 3-*O*-rutinoside, *Am*Del, *Antirrhinum majus* Delila, *Am*Ros1, *Antirrhinum majus* Rosea1, Anthocyanins, Natural colours, Plant cell culture, Blackcurrant

## Abstract

Anthocyanins are widely distributed, glycosylated, water-soluble plant pigments, which give many fruits and flowers their red, purple or blue colouration. Their beneficial effects in a dietary context have encouraged increasing use of anthocyanins as natural colourants in the food and cosmetic industries. However, the limited availability and diversity of anthocyanins commercially have initiated searches for alternative sources of these natural colourants. In plants, high-level production of secondary metabolites, such as anthocyanins, can be achieved by engineering of regulatory genes as well as genes encoding biosynthetic enzymes. We have used tobacco lines which constitutively produce high levels of cyanidin 3-*O*-rutinoside, delphinidin 3-*O*-rutinoside or a novel anthocyanin, acylated cyanidin 3-*O*-(coumaroyl) rutinoside to generate cell suspension cultures. The cell lines are stable in their production rates and superior to conventional plant cell cultures. Scale-up of anthocyanin production in small scale fermenters has been demonstrated. The cell cultures have also proven to be a suitable system for production of ^13^C-labelled anthocyanins. Our method for anthocyanin production is transferable to other plant species, such as *Arabidopsis thaliana*, demonstrating the potential of this approach for making a wide range of highly-decorated anthocyanins. The tobacco cell cultures represent a customisable and sustainable alternative to conventional anthocyanin production platforms and have considerable potential for use in industrial and medical applications of anthocyanins.

## Highlights

• Tobacco cell cultures for customisable anthocyanin production have been developed.
• Differentially decorated and ^13^C-labelled anthocyanins have been produced.
• Anthocyanin yield has been shown to be stable, up to 30 mg per g dry weight.
• Scale-up anthocyanin production in small scale fermenters and purification have been demonstrated.
• The approach is transferrable to other species for production of highly decorated anthocyanins.

## Graphical abstract

We have developed tobacco cell cultures as customisable and sustainable alternatives to conventional anthocyanin production platforms by engineering expression of regulatory genes together with expression of genes encoding side chain decorating enzymes.

**Figure:**
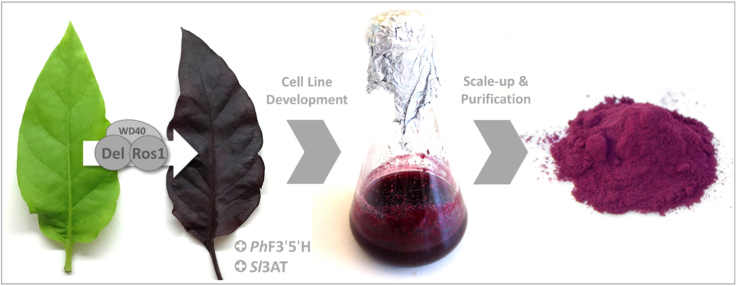

## 1 Introduction

Anthocyanins are a widely distributed group of water-soluble pigments that colour the fruit and flowers of many plants. More than 650 different anthocyanins have been identified, distinguished by methylation, hydroxylation, glycosylation and acylation with both aliphatic and aromatic groups (Andersen and Jordheim, 2010, Zhang et al., 2014). The colour of anthocyanins is influenced by the degree of aromatic acylation, pH, co-pigmentation with other phenolic compounds, metal complexation and can range from orange, through red, pink and purple to blue (Yoshida et al., 2009). Consumption of anthocyanin-rich food promotes health, supported by many recent studies of anthocyanin-rich fruits such as blood orange, blueberry, bilberry and cranberry (Li et al., 2017, Titta et al., 2010, Yousuf et al., 2016), anthocyanin-rich corn (Petroni et al., 2017, Toufektsian et al., 2008) and fruits engineered to be rich in anthocyanins (Butelli et al., 2008, Espley et al., 2014, Zhang et al., 2015). Based on the health-promoting effects of anthocyanins and concerns associated with synthetic food colourants, there is increasing interest in replacement of synthetic dyes by natural anthocyanin colours (Oplatowska-Stachowiak and Elliott, 2017).

The natural colours segment is one of the fastest growing markets of the food and cosmetic colourants industries, with a calculated global market volume for anthocyanins of $291.7 Million in 2014 and a forecast of $387.4 Million for 2021.^3^ Industrial production of anthocyanin pigments relies largely on extraction from whole plants, with the most common sources being waste grape skins from the wine industry, black carrots, red cabbage, sweet potato, and berries (Buchweitz, 2016, Downham and Collins, 2000, Rodriguez-Amaya, 2016). So far, no anthocyanin-based blue colourant is commercially available, and the only source for natural blue colours is phycocyanin from the blue algae, Spirulina (*Arthrospira platensis*, Sigurdson et al., 2017).

The composition of anthocyanins from natural sources varies quantitatively and qualitatively, with the region of origin and the growth season of the plant source (Scalzo et al., 2013, Timmers et al., 2017). Pigment formulations need continued, laborious quality control adjustments to guarantee that extracts have the same colour properties, over time. These restrictions have forced manufacturers to search for alternative sources of natural colourants, with the added attraction of the health-beneficial properties of anthocyanins.

**Fig. 1:**
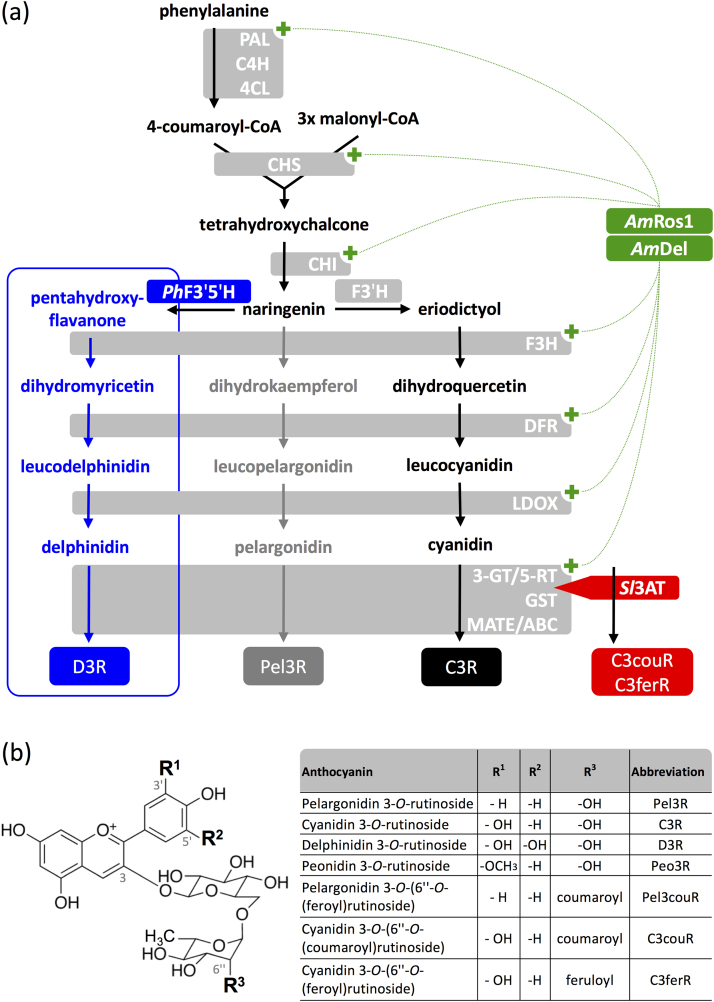
Engineered anthocyanin production in *Nicotiana tabacum*. (a) Anthocyanin biosynthesis pathway. Co-expression of *Am*Del and *Am*Ros1 induces the expression of structural genes (dotted lines) and leads to production of high amounts of cyanidin 3-*O*-rutinoside (C3R) and traces of pelargonidin 3-*O*-rutinoside (Pel3R) in *N. tabacum*. Expression of a *Petunia x hybrida* flavonoid-3′,5′-hydroxylase (*Ph*F3′5′H) leads to hydroxylation of the anthocyanin backbone on the B-ring and production of delphinidin 3-*O*-rutinoside (D3R, blue box). Expression of an anthocyanin 3-*O*-rutinoside-4′′′-hydroxycinnamoyl transferase from *Solanum lycopersicum* (*Sl*3AT) leads to the production of aromatically acylated cyanidin 3-*O*-(6′′-*O*-coumaroyl) rutinoside (C3couR) and cyanidin 3-*O*-(6′′-*O*-feruloyl) rutinoside (C3ferR). (b) Structures of anthocyanins produced in tobacco cultures. Abbreviations: PAL, phenylalanine ammonia-lyase; C4H, cinnamate 4-hydroxylase; 4CL, 4-coumarate-CoA ligase; CHS, chalcone synthase; CHI, chalcone isomerase; F3′H, flavonoid 3′-hydroxylase; F3H, flavanone 3β-hydroxylase; DFR, dihydroflavonol 4-reductase; LDOX, leucoanthocyanidin dioxygenase; GT, glycosyltransferase; GST, glutathione *S*-transferase; MATE, multidrug and toxic compound extrusion transporter; ABC, ATP-binding cassette transporter.

Microbial cell factories have been promoted as anthocyanin production platforms, and allow application of well-established scale-up and bioprocessing technologies for large-scale production (Marienhagen and Bott, 2013). However, such micro-organisms, predominantly yeast and *Escherichia coli* (*E. coli*), require the expression of at least 11 transgenes to introduce the anthocyanin biosynthetic pathway to produce even the simplest anthocyanin, pelargonidin 3-*O*-glucoside, from phenylalanine (Fig. 1). The number of transgenes required increases with the degree of decoration of the target anthocyanins and could easily involve more than 20 transgenes for aromatically acylated anthocyanins with bluish colours. Furthermore, plant cytochrome P450 monooxygenases such as flavonoid 3′ and 3′,5′-hydroxylases (F3′H and F3′5′H), are bottlenecks in microbial production platforms because of inefficient cofactor pools and failures in protein folding and membrane insertion in prokaryotic hosts (Dudnik et al., 2016). Although progress has been made in engineering individual P450 monooxygenases to work efficiently in bacteria (Wei et al., 2018), this requires individual modifications of each P450 protein (Ajikumar et al., 2010) and has not yet been reported for F3′H and F3′5′H for the production of anthocyanins. In contrast to microbes, plant cells contain all the genetic information required to produce and store anthocyanins and, dependent on the species, are not limited to the synthesis of anthocyanin monoglucosides. Commercial use of plant cell cultures was considered early on, because they allow sustainable production in Good Manufacturing Practice (GMP) environments on industrial scales. However, industrial application of plant cell cultures for secondary metabolite production, including anthocyanins, is still rare, mostly as a result of the limited stability of cultures long-term, variable product yields and high costs.

Several strategies have been used to increase anthocyanin yields in plants, mostly through activation of the biosynthetic pathway by ectopic expression of regulatory genes. Anthocyanin biosynthesis is controlled primarily at the transcriptional level by a complex of R2R3MYB, bHLH and WD40 Repeat proteins (the MBW complex) that is well conserved across all angiosperms (Ramsay and Glover, 2005). In the present study, we describe the development of suspension cultures from tobacco plants constitutively expressing the MYB Rosea1 (*Am*Ros1) and bHLH Delila (*Am*Del) transcription factors from *Antirrhinum majus*. These cultures produce exceptionally high levels of anthocyanins. In contrast to cultures generated from non-transgenic anthocyanin-rich tissues, anthocyanin production in these cultures can be maintained stably, independent of the differentiation status of the cells, thus overcoming the obstacle of instability observed in conventional plant cell cultures. To expand the spectrum of different anthocyanins that can be produced, we co-expressed additional transgenes that encode enzymes for further decoration of cyanidin 3-*O*-rutinoside (C3R), the basic anthocyanin produced by tobacco. Concepts for scale-up of cell production and anthocyanin purification to the gram scale have been developed, demonstrating the potential for commercial use in industrial anthocyanin production. We demonstrate that this metabolic engineering approach is applicable to other plant species allowing the production of highly-decorated anthocyanins, which could expand the range of anthocyanin colourants available commercially, especially those towards the blue end of the spectrum.

## 2 Materials and methods

### 2.1 Plant materials and generation of suspension cultures

The generation and genotyping of transgenic tobacco lines have been described in Kallam et al. (2017). All constructs were expressed stably in *Nicotiana tabacum* cv. Samsun under the control of a double *CaMV 35S* promoter in pBin19-derived T-DNAs (Luo et al., 2007). Cell cultures were generated as described in Mustafa et al. (2011) with modifications described in Supplementary Methods and the composition of all media is given in Supplementary Table 1. The *Vitis vinifera* L. cell culture (PC-1137) was obtained from the German Collection of Microorganisms and Cell Cultures (Leibniz Institute DSMZ Braunschweig, Germany) and was maintained in modified B5 medium according to the supplier's instructions (www.dsmz.de/fileadmin/downloads/PC/medium/B5VIT.pdf).

Anthocyanin production from a mutated version of the Delila protein (*Am*Del*) in combination with *Am*Ros1, discovered originally by screening stably-transformed plants carrying 35S:Del and 35S:Ros1 in the same T-DNA for high anthocyanin production without impact on plant growth, was investigated using transient assays in *N. benthamiana* (Northern Territory ecotype (NT), Bally et al., 2015). Infiltration of leaves with *A. tumefaciens* carrying the binary vector for expression of just *Am*Ros1 induced anthocyanin biosynthesis 4 days after infiltration (Supplementary Fig. 1). Infiltration of two *A. tumefaciens* strains expressing *Am*Ros1 and *Am*Del, resulted in about three-fold higher levels of anthocyanin production. Infiltration with *A. tumefaciens* strains carrying vectors for expression of *Am*Ros1 and the truncated version of Delila (*Am*DelΔ) that is encoded by *Am*Del* showed enhanced anthocyanin production compared to *Am*Ros1 alone, but not as great as with unmutated *Am*Del with *Am*Ros1. These results suggested that the stable high level production of anthocyanins produced by 35S:*Am*Ros1: 35S:*Am*Del* results from the N-terminus of the Del protein stabilizing the R2R3Myb protein in its interactions in the MBW complex. Similar reports have been made for deletions of the R/B-proteins in corn in their control of anthocyanin biosynthesis (Goff et al., 1992). We used plants stably transformed with 35S:*Am*Ros1: 35S:*Am*Del* to generate cell cultures for scale-up production of anthocyanins in bioreactors. For the generation of *Arabidopsis thaliana* cultures, Columbia-0 wildtype and *f3′h/tt7-7* plants (Appelhagen et al., 2014) were transformed with the vector carrying 35S:*AmRos1*: 35S:AmDel* through floral dipping. Callus was initiated as described for tobacco and was selected from strongly pigmented upper shoots of young plants. Explants with emerging callus were transferred from induction plates to modified B5 medium (Supplementary Table 1). Darkly pigmented callus was selected until the culture became stable for anthocyanin production. Fruits that were used for anthocyanin quantifications were purchased in a local grocery store.

### 2.2 Anthocyanin labelling with ^13^C-sucrose

Cell cultures of the *Am*Del/*Am*Ros1 line were grown in small scale (20 mL total volume) using 3% (*w/v*) of unlabelled sucrose in the control treatments and 0.5% (*w/v*) fully labelled ^13^C-sucrose (Sigma-Aldrich, Germany, product number 605417) plus 2.5% unlabelled sucrose for anthocyanin labelling experiments. Cells were harvested and freeze dried after 10 days of cultivation at 23 °C. The experiment has been performed twice.

### 2.3 Cultivation of suspension cultures in plant cell bioreactors

Tobacco cells were grown on petri dishes and inoculated in a small amount of nutrient medium (LS supplemented with 1 mg L^−1^ 2.4-D and 100 mg L^−1^ kanamycin). The medium was replaced every seven days, after 3–4 weeks, the suspension cultures were moved to 100 mL culture volume in 1 L Erlenmeyer flasks. For bioreactor cultivation, the LS or tobacco growth medium (Supplementary Table 1), with 1 mgL^−1^ 2,4-D and 100 mgL^−1^ kanamycin was added to the bioreactors prior to sterilization by autoclaving at 121 °C for 15 min. After cooling, the bioreactors were connected to the controller for approximately 24 h prior to inoculation, to stabilize pH and to control and calibrate the flow of O_2_. Upon inoculation, each bioreactor was moved to a flow hood to maintain sterility. Prior to inoculation, the nutrient medium was removed from the Erlenmeyer flasks and the cells were diluted to a concentration of 30 g FW 100 mL^−1^ medium. This suspension was transferred to the bioreactors for a final concentration of 30 g cells L^−1^ medium, or 3% FW in a final volume of 1 L medium. Samples (10 mL) were collected from each reactor every other day, while maintaining constant stirring, to monitor growth rates. The weight of each 10 mL sample was measured, the growth medium was drained and the weight of the remaining cells was measured. The growth rate was calculated from the weight of cells, divided by total weight of the 10 mL sample. The corresponding growth rate per L was then calculated. Nutrient medium was replaced after seven days, and the final harvest was made after 14 days. The samples from each reactor were freeze dried for shipment. All cultures were maintained in the dark.

### 2.4 Anthocyanin extraction, spectrophotometric quantification and LC-UV/MS and LC-MS/MS analysis

Anthocyanins were extracted twice with 500 µL of 80% methanol/10 mg freeze-dried sample, acidified with either 2% formic acid or 1% HCl. The combined extract (1000 µL) was cleared by centrifugation prior to further use. Quantification of total anthocyanin content was performed following the pH differential method described by Giusti and Wrolstad (2001). Total anthocyanin content was calculated based on reference curves for cyanidin 3-*O*-glucoside and cyanidin 3-*O*-rutinoside in the range from 0.001 mg mL^−1^ to 0.05 mg mL^−1^, respectively. For LC-UV/MS analysis, 100 µL of the extract were mixed with 25 µL of solvent A (water + 0.5% formic acid) and incubated for one hour at 4 °C prior to injection to allow the formation of any potential precipitates. After centrifugation, 5 µL of the supernatant were injected per sample. Anthocyanins were analysed by LC-UV/MS as described in Oertel et al. (2017). Details for LC-UV/MS and MS/MS fragmentation analyses are provided in Supplementary Methods. LC-UV/MS data were analysed by means of Bruker Compass DataAnalysis version 4.1 software. The annotation of compounds was based on comparison of the measured retention times and molecular ion masses with reference standards and fragmentation patterns as obtained from LC-MS/MS experiments. Quantification was based on reference curves for the standard compounds C3G and C3R from integrated peak areas of extracted ion chromatograms.

### 2.5 Preparative isolation of anthocyanins from tobacco suspension cultures

Freeze dried cell suspension culture material was ground finely and anthocyanins were extracted sequentially with 80% methanol, 2% formic acid (FA) to a final ratio of 100 mL solvent g^−1^ cell dry weight (DW). After degreasing with heptane and filtering through nylon filters down to 0.2 µm, individual anthocyanins were isolated by sequential chromatographic separation as described in Supplementary Methods. The resulting fractions, showing absorption at 515 nm, were analysed by LC-UV/MS. The volume and the content of organic solvent in the final pure fraction were reduced under vacuum using a rotary evaporator. This fraction was frozen and lyophilised. The dry anthocyanin powder was covered with argon and stored at − 20 °C until further use.

## 3 Results

### 3.1 Suspension cultures of *Am*Del/*Am*Ros1 tobacco plants are a stable source for production of C3R

**Fig. 2:**
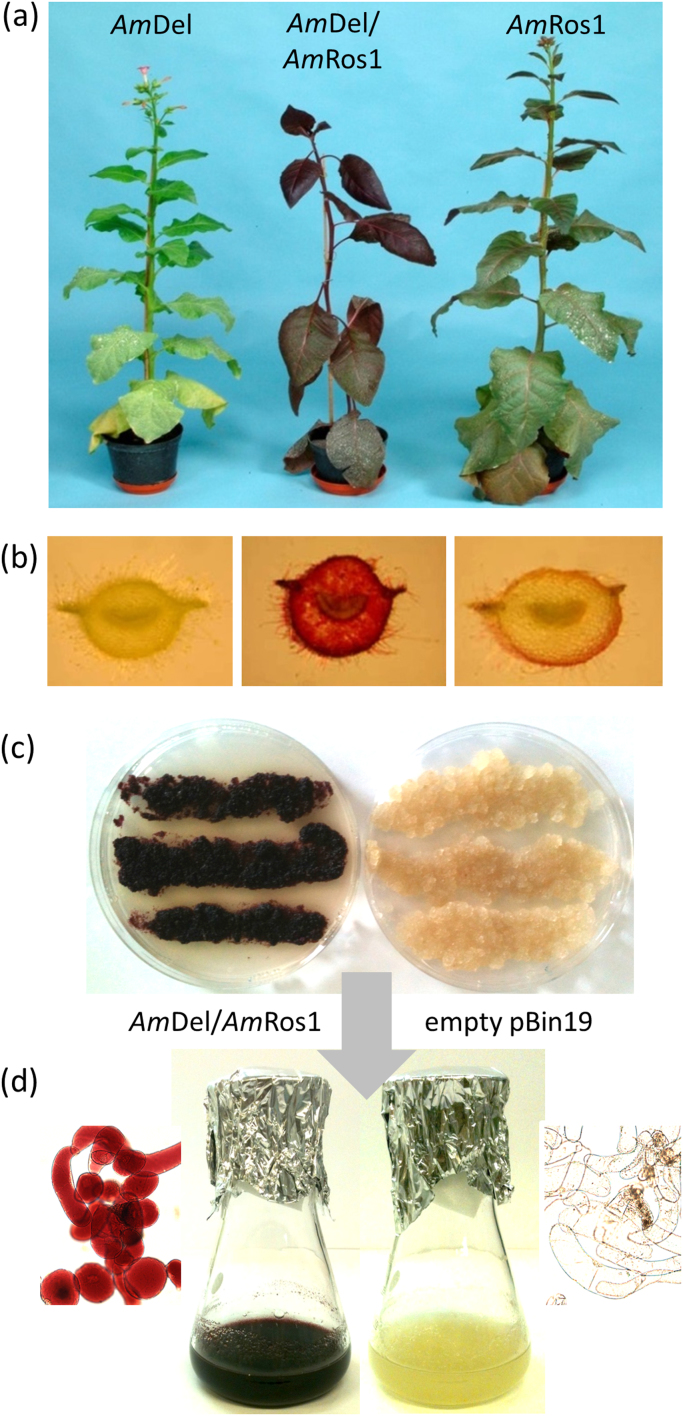
Generation of tobacco suspension cultures. (a) Stably transformed tobacco plants expressing *Am*Del or *Am*Ros1 and both transcription factors together. Only *Am*Del/*Am*Ros1 lines showed strong anthocyanin accumulation throughout the plant. (b) Petiole sections of the same plants. *Am*Del/*Am*Ros1 plants produce anthocyanins in all tissues, whereas in *Am*Ros1 the pigmentation is limited to the epidermis and is completely absent in *Am*Del plants. (c) Dedifferentiated callus from leaf tissue. (d) Suspension cultures from dedifferentiated callus cells in MS medium and microscopic images of cells of suspension cultures.

**Fig. 3:**
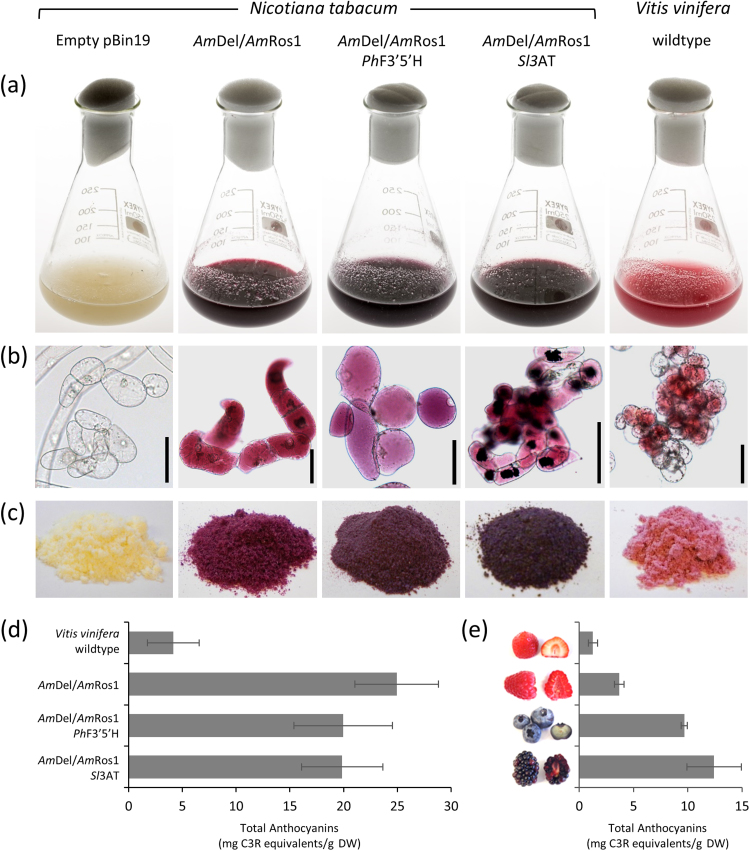
Tobacco suspension cultures. (a) Shake flasks after 7 days of cultivation. (b) Microscopic images of the same suspension cultures. Scale bar 100 µm. (c) Freeze dried powder from suspension cultures. (d) Total anthocyanin amount in different suspension culture batches that have been collected over a period of six months. Error bars show standard deviation of 10 samples. (e) Total anthocyanin content in various fruits, quantified under the same conditions (*n* = 3).

The structural genes of the anthocyanin biosynthetic pathway are usually controlled as a single regulon by MYB-bHLH-WD40 (MBW) transcription factor complexes. Altering the expression of components of the MBW complex has proven to be a powerful tool to manipulate anthocyanin accumulation. We reconstructed anthocyanin biosynthesis in *Nicotiana tabacum* cv. Samsun plants by constitutive co-expression of the *Am*Ros1 and *Am*Del transcription factors from *Antirrhinum majus* (Kallam et al., 2017), Fig. 2a, b). Explants of these lines were dedifferentiated to develop *in vitro* cultures of friable calli, without losing the strength of anthocyanin production (Fig. 2c). Cultures were kept in the dark to prevent the biogenesis of chloroplasts and to maintain the biosynthesis of anthocyanins. Friable calli were subsequently used to establish cell suspensions in liquid MS medium (Fig. 2d), which were grown aerobically and heterotrophically in simple shake flasks with sucrose as carbon source, as described for the BY2 cell line from *Nicotiana tabacum* cv. Bright Yellow 2 (Nagata et al., 1992). Suspensions grew as single cells or as small clusters, which allowed optimal supply of nutrients and oxygen. Anthocyanin accumulation in *N. tabacum* wildtype plants is restricted to flower petals, which produce cyanidin 3-*O*-rutinoside almost exclusively (Luo et al., 2007). Extracts of suspension cultures generated from *Am*Del/*Am*Ros1 plants were analysed by LC-MS and showed accumulation of up to 30 mg C3R g^−1^ DW (Fig. 3d). The oldest *Am*Del/*Am*Ros1 culture has been maintained for more than 10 years without substantial reduction in anthocyanin production, demonstrating its stability, long-term, without aging or silencing of the transgenes.

### 3.2 Tobacco suspension cultures can produce variously decorated anthocyanins

Aiming to produce different anthocyanins with increasing complexity, we additionally expressed a gene encoding flavonoid 3′,5′-hydroxylase from *Petunia x hybrida* (*Ph*F3′5′H) or a gene encoding an anthocyanin 3-*O*-rutinoside-4′′′-hydroxycinnamoyl transferase from *Solanum lycopersicum* (*Sl*3AT) in *Am*Del/*Am*Ros1 tobacco lines (Kallam et al., 2017). Material from these lines was used to generate cell cultures as described for the *Am*Del/*Am*Ros1 lines. All cultures were dark red due to the production of high amounts of anthocyanins, in contrast to a yellowish culture that had been generated from plants carrying the empty pBin19 T-DNA (Fig. 3a-c). In comparison, a conventional cell suspension culture, generated from wildtype grape skins, appeared pale red (Fig. 3a-c, right panel). Cells of the *Am*Del/*Am*Ros1 line were red due to their production of C3R, while cells expressing *Am*Del/*Am*Ros1 and *Ph*F3′5′H were purple due to the production of D3R in addition to C3R. Cultures expressing the tomato anthocyanin 3-acyltransferase produced insoluble anthocyanin precipitates, known as anthocyanic vacuolar inclusions (AVIs, Kallam et al., 2017). In contrast to our anthocyanin tobacco cultures, the conventional grape culture contained sub-populations of cells, which produced no or only very small amounts of anthocyanins, when grown under the same conditions in the dark (Fig. 3b, right panel).

To establish whether the production of anthocyanins was stable over time, we quantified the total anthocyanin content in various batches of tobacco cultures that were grown over a period of six months and compared these to the conventional grape culture (Fig. 3d). All tobacco cultures produced between 4 and 5 times more anthocyanins in total than the grape culture, and had better stability. White cells in the grape culture became more dominant with increasing passages, which made it occasionally necessary to set up new liquid cultures from plate-grown callus, to allow better selection of cells that produced high amounts of anthocyanins. The engineered tobacco cultures, however, were always inoculated with cells from the last batch, and produced between 20 and 25 mg C3R equivalents g^−1^ DW. Compared to the amounts of anthocyanins found in plant tissues, the amount produced in the engineered tobacco cultures was exceptionally high, even when compared to anthocyanin-rich berries like blueberries and blackberries (Fig. 3e).

**Fig. 4:**
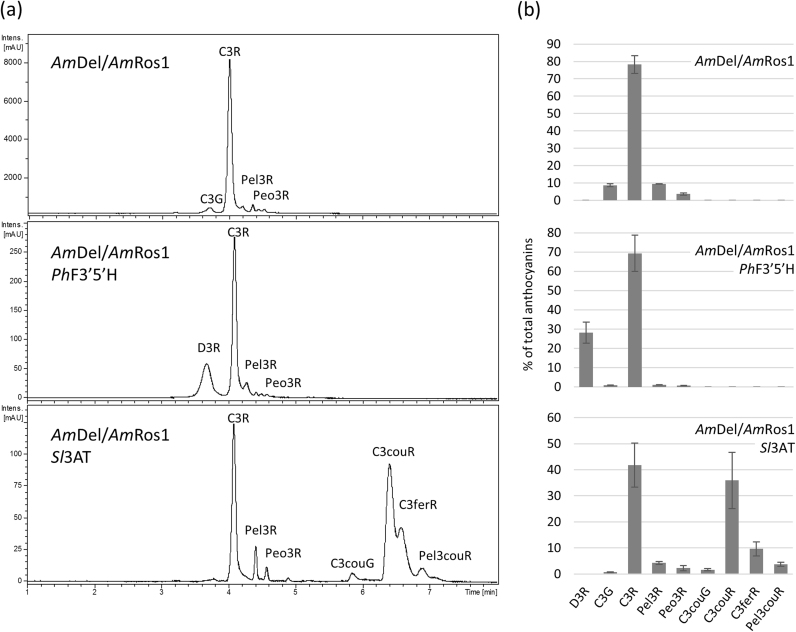
Phytochemical characterisation of anthocyanins produced in tobacco cultures. (a) Representative UV-chromatograms at 515 nm for lines expressing *Am*Del/*Am*Ros1, *Am*Del/*Am*Ros1/*PhF3′5′H* and *Am*Del/*Am*Ros1/*Sl*3AT. (b) Relative amount of anthocyanins, shown as percentage of total anthocyanins calculated from integrated peak areas of respective extracted ion chromatograms. Error bars show standard deviation of 2, 9 and 5 batches. Peak annotation is given in Supplementary Fig. 3.

We analysed the anthocyanin composition in the different tobacco lines and evaluated the relative abundance of different anthocyanins based on the integrated peak areas from extracted ion chromatograms, and identified anthocyanins based on their PDA absorbance and mass fragmentation patterns (Fig. 4, Supplementary Fig. 2, 3 and 4). Anthocyanin extracts of *Am*Del/*Am*Ros1 lines contained almost exclusively C3R, with very minor amounts of cyanidin 3-*O*-glucoside (C3G), pelargonidin 3-*O*-rutinoside (Pel3R) and peonidin 3-*O*-rutinoside (Peo3R). Similar profiles were observed for lines expressing *Am*Del/*Am*Ros1 and *Ph*F3′5′h, except for the additional production of delphinidin 3-*O*-rutinoside (D3R) which made up at least 30% of the total anthocyanins (Fig. 4, middle panel). About half of the anthocyanins in *Am*Del/*Am*Ros1/*Sl*3AT extracts were aromatically acylated with coumaroyl- or feruloyl-moieties, with cyanidin 3-*O*-(coumaroyl) rutinoside (C3couR) and cyanidin 3-*O*-(feruloyl) rutinoside (C3ferR) being the most abundant acylated anthocyanins. Additionally, small amounts of pelargonidin 3-*O*-(coumaroyl) rutinoside (Pel3couR) and cyanidin 3-*O*-(coumaroyl) glucoside (C3couG) were detected (Fig. 4, lower panel). In addition to anthocyanins, we analysed the accumulation of different phenylpropanoids at 280 nm (Supplementary Fig. 5). The most abundant secondary phenylpropanoid compound in all lines was caffeoylquinic acid (CQA), with similar levels between the lines. Interestingly, the pBin19 cell culture without the transcription factors produced only minor amounts of CQA, indicating that *Am*Del/*Am*Ros1 enhances the biosynthesis of chlorogenic acid as well as anthocyanins. As expected, no anthocyanins were detected in cell culture material carrying the T-DNA from a pBin19 empty vector (Supplementary Fig. 5c). Finally, we analysed changes in the anthocyanin and phenylpropanoid composition over time in different batches of the engineered tobacco cultures by LC-MS, without finding major differences (Supplementary Fig. 6). Thus, the production of anthocyanins was stable in the engineered cultures, both quantitatively and qualitatively.

### 3.3 Production of ^13^C labelled anthocyanins

**Fig. 5:**
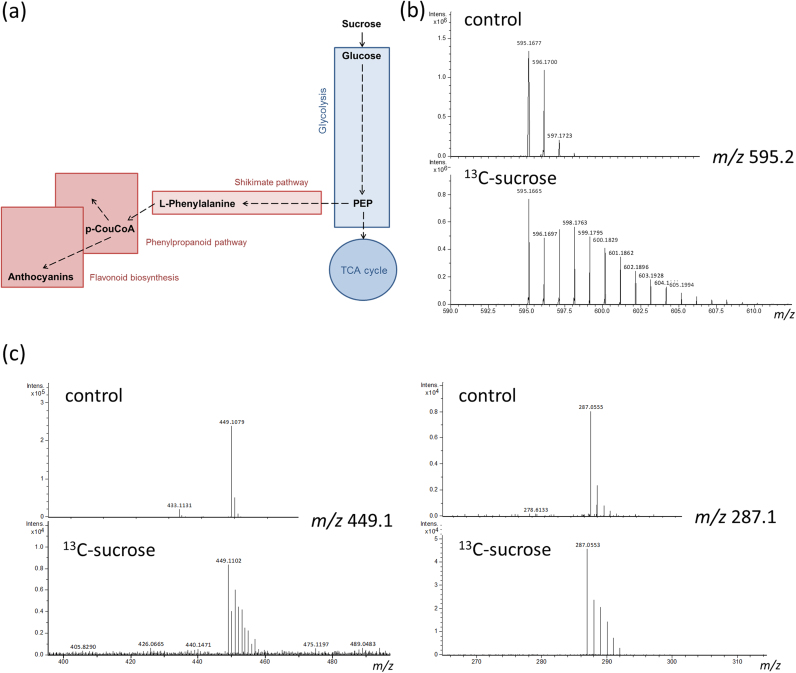
Regioselective ^13^C labelling of anthocyanins. (a) Schematic representation of anthocyanin biosynthesis highlighting major precursors and intermediates. (b) Mass spectrum of C3R showing the isotope pattern under control conditions with unlabelled sucrose (top) and after feeding of ^13^C-sucrose (bottom). The evaluation of the carbon status is given in Supplementary Table 2. (c) Mass spectra of C3G (left) and cyanidin (right) after MS fragmentation of the C3R precursor ion showing the isotope pattern under control conditions (top) and after ^13^C-sucrose treatment (bottom).

Regioselective ^13^C-labelling of anthocyanins can be obtained either by chemical synthesis or by production in plant cells using ^13^C-labelled precursors, such as phenylalanine or sucrose (Fig. 5a). Cultures of the *Am*Del/*Am*Ros1 line were grown with ^13^C-sucrose. Growth was slightly reduced during cultivation with ^13^C-sucrose, but cultures produced similar amounts of C3R to unlabelled cultures. We extracted the sum of mass spectra across the chromatographic peak for C3R (monoisotopic mass *m/z* 595.2) at a retention time of 3.8 min to evaluate the carbon status of both samples (control and ^13^C-sucrose). The mass spectra were compared for their isotopic patterns of C3R (Fig. 5b). Under control conditions six isotopic variants of C3R were detected (Supplementary Table 2, top) although the second and the third isotopic versions accounted for 82% and 16% of the monoisotopic peak, respectively. Higher isotopic variants accounted for only minor percentages (2% for isotope 4, and > 1% for isotope 5 and 6). When one sixth of the sucrose in the medium was replaced by ^13^C-sucrose, the pattern showed a greater number of higher isotopic variants; we were able to detect at least 20 isotopic variants (Supplementary Table 2, bottom). The second and the third isotopic versions accounted for 63% and 71% of the monoisotopic peak, respectively. The fourth isotope accounted for 74% of the monoisotopic peak representing the highest amount of incorporated ^13^C with three atoms per C3R molecule. We concluded that there had been incorporation of three ^13^C molecules in about 70% of the C3R produced. This correlated to the biosynthesis of the anthocyanin backbone from phosphoenolpyruvate (PEP) units (C3-units) leading to an additive effect on the abundance of the fourth isotopic variant of C3R during cultivation with medium containing ^13^C-sucrose. The isotopes 5–11 had intensities between 11% and 64% relative to the monoisotopic peak. Higher isotopic variants were present at percentages below 10%. Incorporation of the ^13^C atoms into the anthocyanin backbone was confirmed by MS fragmentation analysis (Fig. 5c), where the change in isotopic pattern was observed for the C3G fragment ion (*m/z* 449.1) and the cyanidin fragment ion (*m/z* 287.1).

### 3.4 Scale-up in bioreactors

**Fig. 6:**
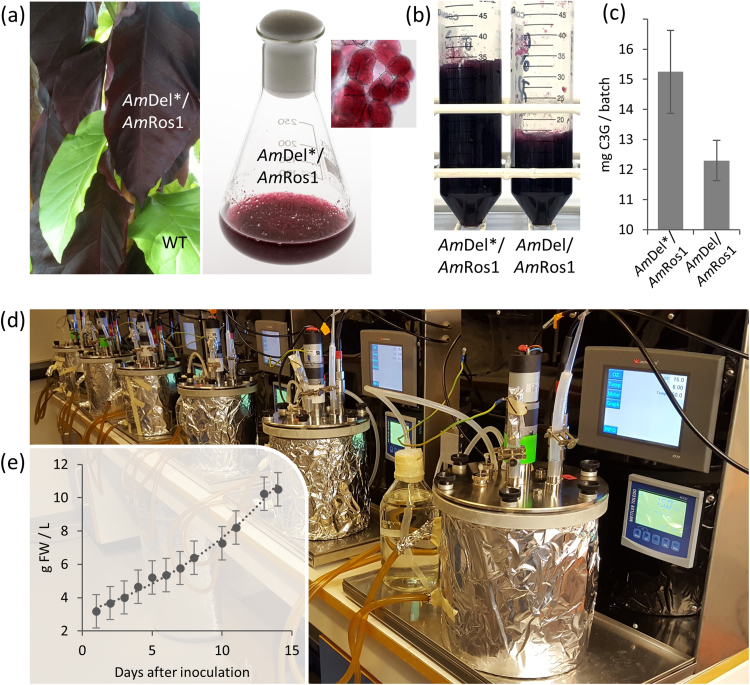
Scale-up in bioreactors. (a) Dark red leaves of an *Am*Del*/*Am*Ros1 tobacco plant next to green leaves of a wildtype plant (left panel) and a suspension culture generated from *Am*Del*/*Am*Ros1 leaves (right panel). (b) Cells harvested from 50 mL suspension cultures after 7 days of growth with the same amount of starting material. (c) Total amount of anthocyanins produced in 50 mL *Am*Del*/*Am*Ros1 and *Am*Del/*Am*Ros1 suspension cultures. Error bar shows standard deviation of anthocyanin yield for 3 batches with each construct. (d) Array of stir tank bioreactors and control units. The outer vessels allow water circulation for temperature control and were wrapped in aluminium foil to prevent light exposure. (e) Growth curves of *Am*Del*/*Am*Ros1 suspensions from four different bioreactor runs, grown in LS medium at 23° after inoculation with 3% packed cell volume. Biomass is shown as average FW ± standard deviation.

The degree of scale-up required to make a production system economically viable depends on the value of the product. The value of pure anthocyanins in today's market is at least $120 mg^−1^, where they are commercially available, meaning that exploring factors that might limit scale-up production to, at least, the 1–10 L level was necessary. The lines carrying 35S:*Am*Del and 35S:*Am*Ros1 were derived by crossing lines homozygous for the individual transgenes, and produced very high levels of anthocyanins but were relatively slow growing, even in shake flasks. To identify lines more suitable for scale-up production of anthocyanins, we selected one primary transformant with a T-DNA insertion from a vector carrying both 35S:*AmDel* and 35S:*AmRos1,* which produced somewhat lower levels of anthocyanin which did not inhibit growth of the plants (Fig. 6a). We investigated why this line supported better plant growth, albeit with lower levels of anthocyanin production compared to the *Am*Del/*Am*Ros1 plants produced from crosses. Molecular analysis showed that this line carried a 4 bp insertion in the sequence encoding *Am*Del, such that a truncated Delila protein was produced, explaining the lower level of anthocyanin production in this line. Callus from these plants initially produced sub-populations of pale or uncoloured cells, but continuous selection of dark calli allowed us to establish a suspension culture that stably produced about 20 mg C3R mg^−1^ DW in shake flasks. Despite lower anthocyanin production, this line (named AmDel*/*AmRos1*) was characterised by fast cell growth and higher net total anthocyanin yields (Fig. 6b, c), consistent with our observation that the production of higher amounts of anthocyanins was associated with limited cell growth, as also reported for strawberry and other plant cell cultures (Deroles, 2009, Nakamura et al., 1998).

We used six small industrial scale bioreactors (2 L, Fig. 6d) specially designed for the cultivation of plant cells (Hvoslef-Eide et al., 2005, Lyngved et al., 2008). Particular problems associated with culturing of plant cells in commercial fermenters include shear-forces and cell growth on the stirring unit and sensors, which were minimised in our customised fermenters. Enrichment of the culture medium with sterile oxygen was achieved through thin-walled (0.2 mm) silicone tubes, in free-hanging loops, which allowed oxygen to pass through, but not the normal contaminants of laboratory air, which prevented infections while ensuring an optimal concentration of oxygen for the rapid proliferation of plant cells (Heyerdahl et al., 1995). We transferred the growth of *Am*Del*/*Am*Ros1 line from shake flasks to these bioreactors. Several inoculation levels (2%, 3%, and 5%) were tested in LS medium, defining an optimal concentration of 3% starting material for this cell line. Optimum growth rates were obtained at 23 °C. We cultivated cells in parallel in four bioreactors and monitored growth over 14 days (Fig. 6e). Phytochemical characterisation did not show any differences in the anthocyanin composition when compared to flask-grown cultures. With tobacco growth medium with lowered 2,4-D concentration, the total amount produced in the bioreactors was up to 90 mg C3G equivalents L^−1^ culture medium or 180 mg per bioreactor run. At today's prices we estimate this to have a value of > $20,000.

### 3.5 Purification of anthocyanins from plant cell cultures to analytical standard grade

**Fig. 7:**
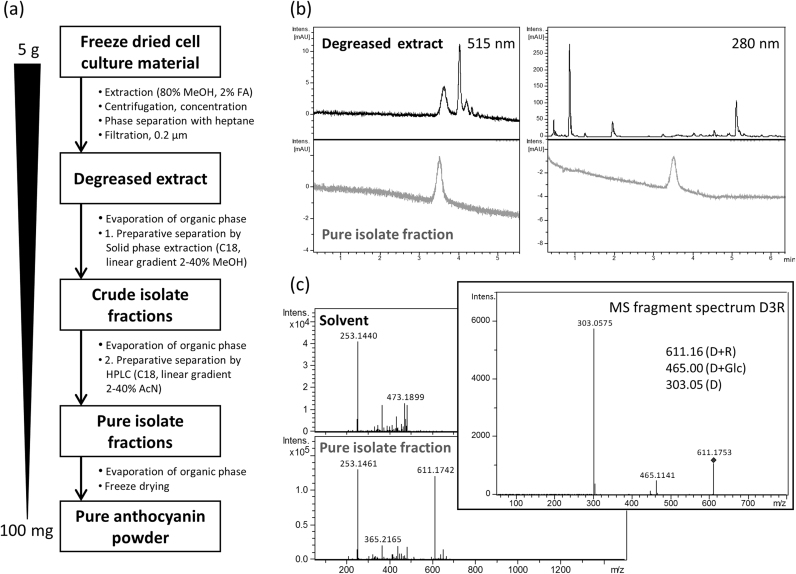
Anthocyanin purification from tobacco cell suspension cultures. (a) Purification scheme. (b) Representative UV-chromatograms at 515 nm and 280 nm for defatted extract and pure isolate fractions from purification of D3R. (C) Purity check by mass spectrometry analysis. MS spectra of solvent and pure isolate fractions are shown together with the MS fragmentation spectrum for confirming the identity of the isolated D3R. Abbreviations: delphinidin (D); glucoside (Glc); rutinoside (R); delphinidin 3-*O*-rutinoside (D3R); methanol (MeOH); acetonitrile (AcN).

We developed a purification pipeline to isolate pure anthocyanins from the tobacco cultures. The overall purification strategy is shown in Fig. 7a together with results from the purification of D3R from *Am*Del/*Am*Ros1/*Ph*F3′5′H cultures. When using solvents containing hydrochloric acid (HCl) we observed rapid hydrolysis of conjugated sugars and browning due to degradation of anthocyanins. Therefore, we used methanol acidified with formic acid for extraction. Extracts generated this way were stable in colour, total anthocyanin amounts and composition for a period of several weeks when stored at 4 °C. To remove soluble lipophilic compounds from the crude extracts, a two-phase separation by the non-polar solvent, *n*-heptane was included. Micro-particles were removed by filtration through nylon membranes giving a clear polar extract with a comparable chromatographic profile (Fig. 7b, upper panel) to those obtained from the simple cell extracts. Consequently, we used column chromatography separation on C18-derivatized silica matrices because this offered the possibility of gradient elution and automation of UV-monitoring and fraction collection. Column separation removed the main non-anthocyanin phenylpropanoids, primarily CQA, and generated fractions containing anthocyanins, even the minor ones. For final preparative liquid chromatography purification, a C6 bonded phenyl ligand (Phenyl-Hexyl) based matrix was used because it gave unique selectivity for aromatic and moderately polar analytes through pi-pi interactions. As a result, highly pure anthocyanin preparations were obtained (Fig. 7b, lower panel) with UV-chromatogram-based purities of 95–98% and mass spectrometry based purities of 70–78%. The identities of the purified anthocyanins were confirmed by MS fragment analysis (Fig. 7c). The combination of these methods gave recovery rates of about 90%.

### 3.6 Engineered cell cultures of different species can produce anthocyanins with multiple decorations

**Fig. 8:**
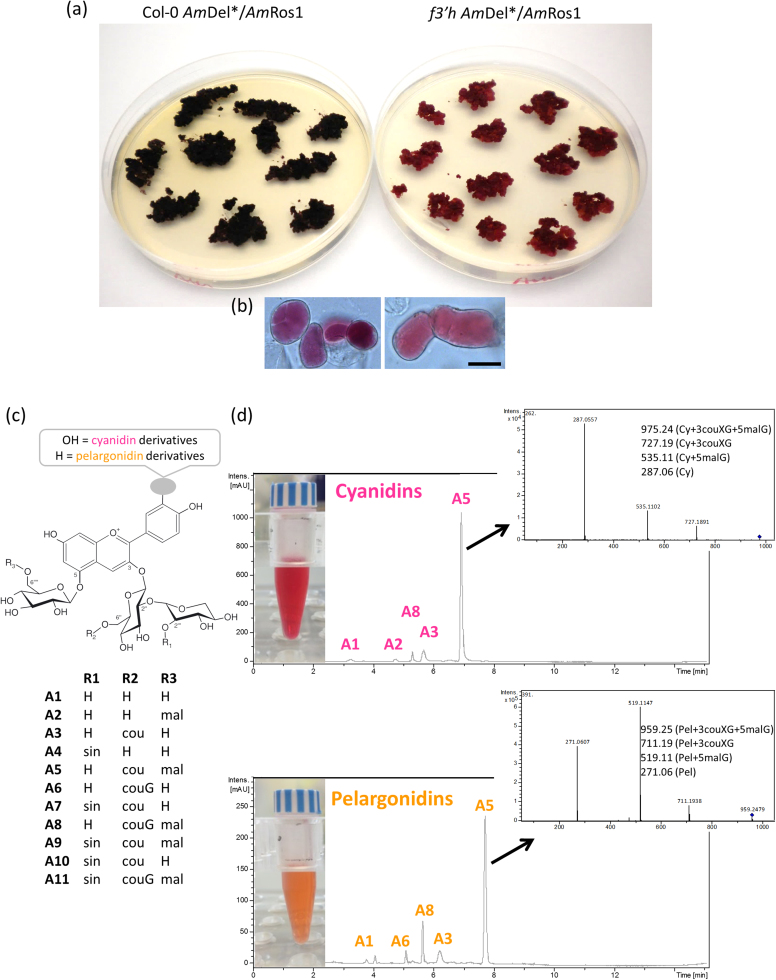
Anthocyanins from Arabidopsis cell cultures. (a) Callus cultures generated from Col-0 (left) and *f3′h* plants (right), both expressing *Am*Del*/*Am*Ros1. (b) Microscopic images of cells from the same cultures. Scale bar 25 µm. (c) Anthocyanin structures detected in *A. thaliana*. (d) UV chromatograms at 515 nm and MS fragmentation pattern of extracts from cultures carrying the *Am*Del*/*Am*Ros1 construct in Col-0 (upper panel) and in the *f3′h* background (lower panel). The major compounds are cyanidin 3-*O*-[2′′-*O*-(xylosyl)−6′′-*O*-(*p*-coumaroyl) glucoside] 5-*O*-malonylglucoside (*m/z* 975.24) and pelargonidin cyanidin 3-*O*-[2′′-*O*-(xylosyl)−6′′-*O*-(*p*-coumaroyl) glucoside] 5-*O*-malonylglucoside (*m/z* 959.25).

**Fig. 9:**
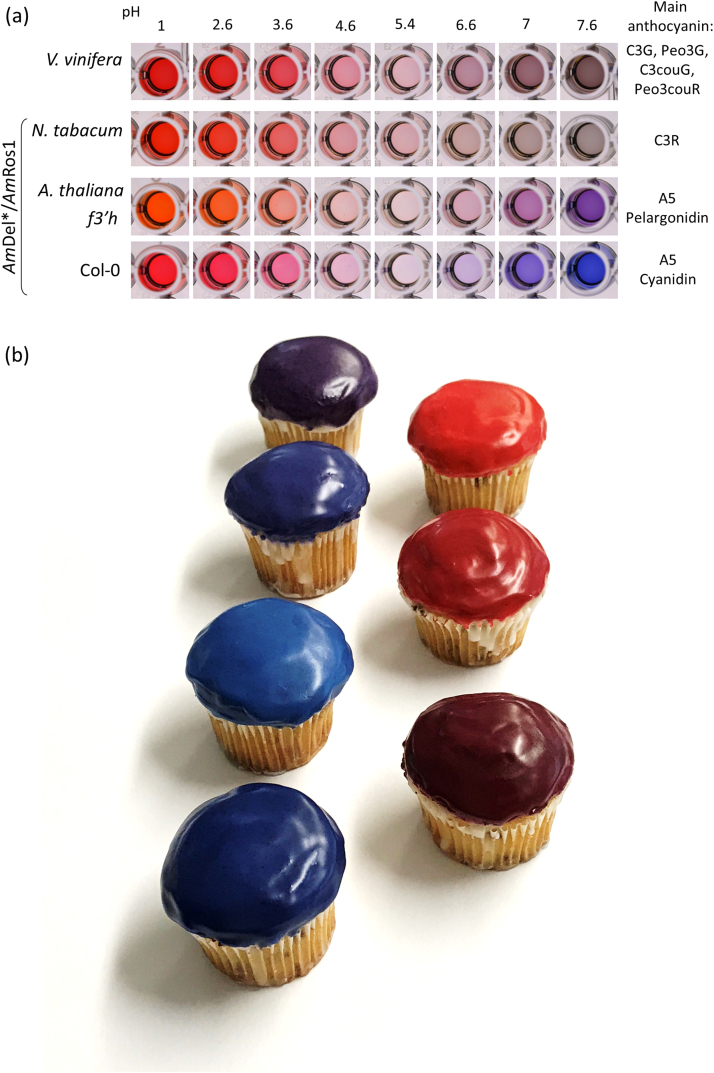
Application of anthocyanins from plant cell cultures as food colourants. (a) Colours of anthocyanin extracts (200 µM C3R equivalents) from *Am*Del*/*Am*Ros1 Arabidopsis cultures in 25 mM KCL pH1 and McIlvaine's citrate-phosphate buffer at various pH, as indicated, in comparison to extracts of AmDel*/*Am*Ros1 tobacco cultures and a conventional grape culture. (b) Cupcakes decorated with icing sugar, supplemented with anthocyanins from Arabidopsis *Am*Del*/*Am*Ros1 cell cultures. Preparations with acylated cyanidins had purple to dark blue colours at neutral and slightly alkaline pH (cupcakes on the left-hand side) and aubergine hues at acidic pH (lower cupcake on the right-hand side). Icing with extracts containing acylated pelargonidins had orange to red colours at acidic pH (cupcakes on the right-hand side).

There is considerable commercial interest in developing strong blue anthocyanin preparations and to improve the stability of anthocyanins such that they can be used industrially as reliable, natural colourants. The amounts and types of anthocyanins that we have been able to generate from tobacco cell cultures are currently dominated by anthocyanins with red to purple colours (Fig. 3, Fig. 9). To establish whether our engineering strategy could be transferred to plants which produce more highly decorated anthocyanins, especially those that give rise to bluer colours, we generated cell cultures from *Arabidopsis thaliana*, aiming at the production of anthocyanins with multiple acyl-groups (Fig. 8). Callus cultures were generated from Col-0 and *f3′h* plants, both expressing the *Am*Del*/*Am*Ros1 transcription factors. Arabidopsis *f3′h* mutants can produce only pelargonidin-derived anthocyanins, due to the lack of 3′-hydroxylation of the anthocyanin B-ring (Appelhagen et al., 2014, Fig. 1). Cells of the Col-0 culture appeared dark purple due to the accumulation of acylated cyanidin derivatives while cells of the *f3′h* culture had a more coral colour due to accumulation of acylated pelargonidin derivatives (Fig. 8a, b). A11, the most abundant and most highly modified anthocyanin in Arabidopsis, is decorated with four glycosyl and three acyl-groups (cyanidin 3-*O*-[2′′-*O*-(2′′′-*O*-(sinapoyl) xylosyl) 6′′-*O*-(*p*-*O*-(glucosyl)-*p*-coumaroyl) glucoside] 5-*O*-[6′′′′-*O*-(malonyl) glucoside], Bloor and Abrahams, 2002). The other anthocyanins, A1-A10, represent less decorated precursors of A11. Analysis of the anthocyanins produced by the Arabidopsis cultures showed cyanidin 3-*O*-[2′′-*O*-(xylosyl)−6"-*O*-(*p*-coumaroyl) glucoside] 5-*O*-[6′′′′-*O*-(malonyl) glucoside] to be the major anthocyanin in Col-0 lines (*m/z* 975.24) and pelargonidin 3-*O*-[2′′-*O*-(xylosyl)−6′′-*O*-(*p*-coumaroyl) glucoside] 5-*O*-[6′′′′-*O*-(malonyl) glucoside] to be the major anthocyanin in the *f3′h* lines (*m/z* 959.25, Fig. 8d). Both anthocyanins lack the sinapoyl and glucosyl moieties attached to the glucose on the C3 position and correspond to the A5 type anthocyanin found in wildtype Arabidopsis Col-0 (Fig. 8c).

The colour of anthocyanins at higher pH is of particular interest because many food and cosmetic applications involve environments of pH 6–8. The colours of the anthocyanins from Arabidopsis were compared at various pH to anthocyanins extracted from a conventional grape culture and a C3R tobacco culture (Fig. 9a). The anthocyanin extracts from Arabidopsis were purple to blue at neutral and slightly alkaline pH, whereas both the tobacco anthocyanin and those from the grape culture, had a brown grey hue, which quickly faded away. To demonstrate that these anthocyanins can also exhibit blue colours in food applications, we prepared cupcakes with icing that had been supplemented with freeze-dried powder preparations of our Arabidopsis cultures (Fig. 9b). By changing the pH with citrate-phosphate buffer, we generated purple colours and different shades of blue at neutral and slightly alkaline pH with the acylated Arabidopsis cyanidins, and orange to red colours at acidic pH with the acylated cyanidins and pelargonidins, respectively.

## 4 Discussion

### 4.1 Anthocyanin production in *Am*Del/*Am*Ros1 cell cultures

Currently, the main commercial sources of anthocyanins are crude extracts from fruits or waste products from the beverage industries. These are not standardised with respect to the particular anthocyanins they contain, nor the amounts of each anthocyanin in the extract. The production in *Am*Del/*Am*Ros1 cell cultures in contrast allowed us to control the type of anthocyanin produced and increased yields substantially (Fig. 3d). Our tobacco cultures produced C3R after activating the anthocyanin biosynthetic pathway with the *Am*Del/*Am*Ros1 transcription factors and D3R or C3couR after expression of *Ph*F′5′H and *Sl*3AT, respectively (Fig. 4). C3R and D3R are the major anthocyanins in blackcurrant and other berry juices (Nielsen et al., 2003). The anthocyanin composition in extracts of these cultures is simple, with just one or two major anthocyanins, which allows easy purification of pure compounds (Fig. 4, Fig. 7). The cultures produced the same anthocyanins as the corresponding tobacco plants (Kallam et al., 2017), with the exception of small quantities of Peo3R (less than 4% of the total anthocyanins), presumably through the activity of endogenous methyltransferases (Nakamura et al., 1998, Zhou et al., 2012).

The number of commercially available pure anthocyanins is very small and limited to mono- and diglycosides of cyanidin, delphinidin, peonidin and petunidin, while acylated anthocyanins are almost entirely unavailable. The repertoire of anthocyanins produced in *Am*Del/*Am*Ros1 cell cultures can be expanded, as demonstrated by the production of D3R and C3couR. The co-expression of different glycosyl- and acyltransferases can be used as a molecular toolkit to produce variously decorated custom-made anthocyanins (Kallam et al., 2017). Acylated anthocyanins are of particular interest, due to their increased stability and for aromatic acylation, their colour properties. Aromatic acylation causes a bathochromic shift such that the absorption maximum of the anthocyanin is shifted towards red and the pigment appears bluer (Luo et al., 2007). Some of the most intense blues in flowers (such as those in morning glory and delphinium) are conferred by highly acylated anthocyanins (Yoshida et al., 2009). We extended our approach by expressing *Am*Del*/*Am*Ros1 in Arabidopsis, which already carries the genetic information to produce tri-acylated anthocyanins. The decoration-pattern of Arabidopsis anthocyanins depends on the growth conditions and stimuli that were used to induce anthocyanin biosynthesis (Kovinich et al., 2014). The most decorated and usually most abundant anthocyanidin in Arabidopsis wildtype leaves is the coumaroylated, sinapoylated and malonylated cyanidin A11 (Bloor and Abrahams, 2002). The Col-0 and *f3′h Am*Del*/*Am*Ros1 cultures of Arabidopsis produced predominantly A5-type anthocyanins without the sinapoyl- and second glucosyl-groups on the anthocyanins (Fig. 8). Similar anthocyanin profiles have been described for cultured cells and roots of the Arabidopsis *pap1-D* mutant (Tohge et al., 2005, Zhou et al., 2012), that ectopically produces anthocyanins as a result of insertion of a T-DNA for activation tagging close to the *PAP1* (*MYB75*) gene. Anthocyanin acyltransferases belong to two different families. BAHD acyltransferases localise to the cytoplasm and use acyl-coenzyme A thioesters as acyl-donors, while serine carboxypeptidase-like (SCPL) acyltransferases are vacuolar enzymes that utilise acyl-sugars as substrates (Sasaki et al., 2014). The Arabidopsis anthocyanin 3-*O*-glucoside coumaroyltransferase and anthocyanin 5-*O*-glucoside malonyltransferase are BAHD acyltransferases required for the synthesis of A5 (Luo et al., 2007), while the anthocyanin 3-*O*-glucoside-2″-*O*-xyloside: sinapoyltransferase (SCLP10) belongs to the SCLP family (Fraser et al., 2007). A lack of sinapoylated anthocyanins has also been reported after loss of SCLP10 activity and for *ugt84a2* mutants, suggesting that UGT84A2 is crucial for the supply of 1-*O*-sinapoyl glucose for the biosynthesis of A11 (Yonekura‐Sakakibara et al., 2012). Similarly, the supply of sinapoyl glucose may limit the production of sinapoylated anthocyanins in our *Am*Del*/*Am*Ros1 cultures or the expression of *SCLP10* may not be activated by the transcription factors. When compared to A11, the final glycosylation of the coumarate moiety is also absent in A5, which, like sinapoylation, is catalysed by a vacuolar enzyme, the acyl-glucose dependent glycosyltransferase BGLU10 (Miyahara et al., 2013). In contrast to the SCLP catalysed steps, both cultures, Col-0 and *f3′h*, produced A8-type anthocyanins, possibly due to some BGLU10 activity. However, we cannot rule out that the expression of the vacuolar acyl- and glycosyltransferases is not under control of *Am*Del*/*Am*Ros1. Induction of these genes might require a full-length bHLH or different regulators. Interestingly, overexpression of PAP1 preferentially increased accumulation of A5 (208-fold induction compared to controls) relative to A11 (29.31-fold) suggesting that even the endogenous MBW complex does not activate the SCLP10 and BGLU10 as strongly as other genes in the anthocyanin biosynthetic pathway (Tohge et al., 2005). The expression of the anthocyanin BAHD acyltransferases and UDP-sugar dependent glycosyltransferases (UGTs) is controlled by the MBW complex, as already demonstrated in tomato (Butelli et al., 2008, Tohge et al., 2015, Zhang et al., 2014) and by the induced production of A5 in our Arabidopsis cell cultures (Fig. 8). The production of anthocyanins through expression of *Am*Del*/*Am*Ros1 and without further engineering of vacuolar glycosyl- and acyltransferases might be limited to anthocyanins with decorations added by BAHD acyltransferases and UGTs.

Blue anthocyanins are industrially important, due to the lack of natural blue colourants and the desire to replace synthetic food dyes with natural pigments (Sigurdson et al., 2017). The best targets for stable blue colours are aromatically acylated anthocyanins. The acylated A5 cyanidin from the Col-0 Arabidopsis culture showed improved stability of blue colours at neutral to slightly alkaline pH, whereas the non- or monoacylated cyanidin and peonidin glycosides from the tobacco and grape cultures had almost no colour at these pH values and gave browning upon oxidation (Fig. 9). The Arabidopsis A5 cyanidin confirms the idea that acylated anthocyanins could provide a blue pigment suitable as a replacement for synthetic blue dyes.

### 4.2 Production of ^13^C-labelled anthocyanins in *Am*Del/*Am*Ros1 cell cultures

Despite the high demand of labelled anthocyanins for bioavailability and tracer studies in humans, regioselective ^13^C-labelling of anthocyanins has been shown only rarely. Production of ^13^C-labelled anthocyanins by *Vitis vinifera* cell suspension cultures was demonstrated decades ago (Krisa et al., 1999), but the system never went into commercial use. Recent studies on the pharmacokinetics of anthocyanins and their metabolites in humans (Czank et al., 2013, Ferrars et al., 2014) relied on chemical synthesis of the isotopically labelled anthocyanin, C3G (Zhang et al., 2011). We have shown the suitability of our cell cultures for the ^13^C-labelling of anthocyanins. The observed reduction in cell growth upon addition of ^13^C-sucrose may be overcome by application of the ^13^C-labelled precursor after exponential growth of the cells has started, as shown for ^13^C-phenylalanine application in *Vitis vinifera* cell suspension cultures (Krisa et al., 1999). Full labelling of the anthocyanin backbone would require several rounds of media exchange until the cellular pool of ^13^C-sucrose has reached equilibrium. To date, a maximum ^13^C enrichment of 65% for anthocyanins from *Vitis vinifera* cell suspension cultures (Krisa et al., 1999) and of nearly 100% for chemically synthesised ^13^C_5_-C3G (Zhang et al., 2011) have been reported. Against this background, our cell cultures present a valuable system for the production of defined isotopically labelled anthocyanins which can be used for bioavailability and degradation studies in humans.

### 4.3 Plant cell cultures versus microbial production platforms

Flavonoids such as anthocyanins are made specifically by plants and microbes such as yeast and *E. coli* require extensive engineering to produce anthocyanins. The main hurdles are the cytochrome P450 monooxygenases F3′H and F3′5′H, which have low activity in prokaryotic hosts. When anthocyanins are synthesised from coumaroyl-CoA and malonyl-CoA in bacterial hosts, production is limited to pelargonidin derivatives with just one hydroxyl group on the B-ring, due to the low activity of the F3′H and F3′5′H cytochrome P450 proteins. Circumvention of these limiting steps has been achieved by feeding precursors to produce cyanidin- and delphinidin-derived anthocyanins. Progress has been made in engineering anthocyanin production in *E. coli* by feeding of eriodictyol or catechin and their conversion to cyanidin 3-*O*-glucoside (Chouhan et al., 2017). Production titers in the mg L^−1^ range have been obtained after improving substrate uptake, increasing the intracellular availability of UDP-glucose, inhibition of the UDP-glucose degradation pathway and construction of artificial enzyme clusters to avoid fast degradation of anthocyanidin aglycones (Leonard et al., 2008, Lim et al., 2015, Yan et al., 2008). Plant cell cultures, in contrast, can produce relatively large amounts of anthocyanins from just sugar, without precursor-feeding or extensive engineering of enzymes to improve metabolic flux, and without the production of non-specific side-products. More recently an *E. coli* polyculture strategy has been developed to synthesise pelargonidin 3-*O*-glucoside (Pel3G) from *p*-coumaric and caffeic acids derived from different sugars (Jones et al., 2017). Although encouraging, these protocols have not been demonstrated as effective under scale-up conditions where competition between different microbial components of the communities might limit production of anthocyanins. Similar approaches have been taken for anthocyanin production in *Saccharomyces cerevisiae*, without substantial success in high level production of anthocyanins (Krivoruchko and Nielsen, 2015).

The three pillars of efficient production platforms are product titers, total yield, and production rate. About 25–30 mg per g DW of *Am*Del/*Am*Ros1 cultures consists of anthocyanins, which makes their yield superior to other production platforms that synthesise anthocyanins from sugars. *De novo* synthesis in *E. coli* polycultures has led to the production of up to 10 mg Pel3G L^−1^ (Jones et al., 2017), in comparison to 90 mg C3R L^−1^ in our plant cell bioreactor experiments, which is at least nine times higher yields. However, plant cells grow more slowly than *E. coli* and, consequently, must have higher yields to match the production rates of microbial systems. Preliminary experiments with improved growth medium indicated that yield in our tobacco cultures can be increased substantially under current scale-up conditions, making the plant cell production platforms comparable to microbial ones. Microbial systems might be suitable platforms for the production of simple pelargonidin glycosides or, with precursor-feeding, for cyanidin and delphinidin glycosides, but the main strength of plant cell cultures is their ability to produce rare anthocyanins with highly decorated side-chains that are currently unavailable, commercially. The activation of the whole anthocyanin pathway simply by expressing the *Am*Del/*Am*Ros1 transcription factors allows rapid development of new cell lines, particularly when existing cell cultures can be transformed directly, and offers flexibility in the production of different anthocyanins, with properties widely diverged from those of simple anthocyanin glycosides

### 4.4 Are the *Am*Del/*Am*Ros1 cultures superior to non-transformed, non-GM cultures?

Commercial use of plant cell cultures for the production of high-value secondary metabolites, such as terpenoids, flavonoids and alkaloids has been considered for many decades (Ananga et al., 2013, Davies and Deroles, 2014, Nosov, 2012, Ochoa-Villarreal et al., 2016, Roberts, 2007, Seitz and Hinderer, 1988, Wilson and Roberts, 2012). Cell cultures for the production of anthocyanins have been developed from black carrot (Gläßgen et al., 1992), grape skins (Ananga et al., 2013, Fig. 3), sweet potato (Konczak-Islam et al., 2003, Nozue et al., 1987) and other plants (Deroles, 2009), but no cell culture system has yet made it to bulk production on industrial-scales. Plant cells cultures are typically heterogeneous in their cell composition, as a result of selection during callus formation and de-differentiation, which can cause variation in growth rates and product yields (Ochoa-Villarreal et al., 2016). Culturing cambial meristematic cells, the stem cells that form vascular tissues, is a promising alternative to dedifferentiated cells, with better growth rates, aggregate sizes and overall consistency (Lee et al., 2010, Ochoa-Villarreal et al., 2016). However, it is unlikely that anthocyanin biosynthesis can be induced in these cells without genetic manipulation because anthocyanin biosynthesis does not normally occur in cambial or vascular tissues. The major limitation in conventional cell cultures is that anthocyanins, similar to most natural compounds, accumulate in specialised differentiated cells, but fast and continuous growth of suspension cultures requires undifferentiated cells. Dedifferentiation is usually associated with loss of anthocyanin production (Deroles, 2009). Conventional cell cultures from wildtype grapes produced anthocyanins mainly in small cell aggregates that underwent some level of differentiation (clearly recognisable by their regular spherical shape, Fig. 3b), while the fast-growing undifferentiated fraction of cells lost their ability to produce anthocyanins (Ananga et al., 2013, Fig. 3). Cells that do not produce anthocyanins usually have shorter doubling times and consequently become dominant over time and cause instability of these cultures. The conflict between growth rate and anthocyanin production requires the maintenance of a differentiation state that is an effective balance between the two extremes (Deroles, 2009). Plant materials must be continuously selected and cryobanks used to source materials for the growth of production batches. This is expensive and the resulting production costs are often too high to match sales-prices, particularly in the natural colourants market. In contrast to anthocyanins, some active pharmaceutical ingredients with limited availability and very high value have been produced economically in cell cultures such as the diterpenoid chemotherapeutic agents, Paclitaxel and Docetaxel, both produced in cell cultures of *Taxus brevifolia* (Howat et al., 2014).

The expression of the *Am*Del/*Am*Ros1 transcription factors activates anthocyanin biosynthesis independent of the cell type and leads to anthocyanin accumulation in all cells (Fig. 3b). The total yields are higher than in conventional cultures. The continuous anthocyanin accumulation in *Am*Del/*Am*Ros1 cultures simplifies and reduces the time that is required to develop novel cell cultures, because selection of anthocyanin-producing cells with the right differentiation state is unnecessary. Silencing of the transgenes can be prevented by selection with kanamycin, which, as a positive side effect, also prevents microbial contamination. The constitutive expression of *Am*Del/*Am*Ros1 avoids aging of the cell cultures and confers long-term stability. We found that most of our *Am*Del/*Am*Ros1 lines produced exceptionally high amounts of C3R, but grew slowly when compared to the pBin19 control or BY2 cell line, depending on the amounts of anthocyanins produced. This is consistent with previous observations that anthocyanin production affects cell growth reciprocally (Deroles, 2009). Efficient production systems require a good balance between anthocyanin production and cell growth to maximise yields. Using the truncated Delila protein in the *Am*Del*/*Am*Ros1 lines, we found a simple way to reduce anthocyanin production to increase biomass production (Fig. 6a-c), in multiple plant hosts.

Production costs are crucial for commercialisation of plant cell cultures, particularly for colourants and pigments with lower market values than fine chemicals or pharmaceuticals. The easy and rapid development and the very high yields together with the stability of our cell cultures offer the potential to reduce the costs of anthocyanin production substantially. The system is flexible and can be set up in a relatively short time for different species, as demonstrated for the diacylated anthocyanins from Arabidopsis cultures (Fig. 8). Since anthocyanins with multiple aromatic acyl-groups are promising targets to replace synthetic blue food colourants, production in cell cultures of GRAS (Generally Regarded As Save) species, such as tomato and potato, could be of particular interest. This approach could also minimise the inclusion of unwanted by-products such as allergens and toxins in coloring food additives and potentially allows the use of crude extracts of cell cultures as colourants without the need for extensive and costly purification.

While the beneficial attributes of these cultures of necessity involve genetic modification, this is equally true of all microbial production systems. Regulatory approval for food colourant applications would involve cell cultures from GRAS species and demonstration that the effects of the individual transformation events are safe for human consumption.

## 5 Conclusion

Anthocyanin production is stable in *Am*Del/*Am*Ros1 cultures. They have higher yields than conventional cultures which can reduce production costs substantially. The expression of *Am*Del/*Am*Ros1 can be extended to other species, which already carry the genetic information to produce exotic anthocyanins, such as variously acylated anthocyanins. Cell cultures, also in bioreactors, offer an easy tool to expand the potential range of commercially available anthocyanins, on different scales, either as high-value fine chemicals or for the bulk production of colourants as well as for the customised production of labelled anthocyanins which are required for human stable-isotope tracer and bioavailability studies.

## Footnotes

3 http://www.transparencymarketresearch.com/anthocyanin-market.html.

## References

[bib1] Ajikumar P.K., Xiao W.H., Tyo K.E., Wang Y., Simeon F., Leonard E., Mucha O., Phon T.H., Pfeifer B., Stephanopoulos G. (2010). Isoprenoid pathway optimization for Taxol precursor overproduction in Escherichia coli. Science.

[bib2] Ananga A., Phills B., Ochieng J., Georgiev V., Tsolova V., Sladonja B. (2013). Production of anthocyanins in grape cell cultures: a potential source of raw material for pharmaceutical, food, and cosmetic industries. The Mediterranean Genetic Code - Grapevine and Olive.

[bib3] Andersen Ø.M., Jordheim M., Andersen Ø.M., Jordheim M. (2010). Anthocyanins. Encyclopedia of Life Sciences.

[bib4] Appelhagen I., Thiedig K., Nordholt N., Schmidt N., Huep G., Sagasser M., Weisshaar B. (2014). Update on transparent testa mutants from *Arabidopsis thaliana*: characterisation of new alleles from an isogenic collection. Planta.

[bib5] Bally J., Nakasugi K., Jia F., Jung H., Ho S.Y., Wong M., Paul C.M., Naim F., Wood C.C., Crowhurst R.N., Hellens R.P., Dale J.L., Waterhouse P.M. (2015). The extremophile *Nicotiana benthamiana* has traded viral defence for early vigour. Nat. Plants.

[bib6] Bloor S.J., Abrahams S. (2002). The structure of the major anthocyanin in *Arabidopsis thaliana*. Phytochemistry.

[bib7] Buchweitz M., Carle R., Schweiggert R. (2016). Natural solutions for blue colors in food. Handbook on Natural Pigments in Food and Beverages.

[bib8] Butelli E., Titta L., Giorgio M., Mock H.P., Matros A., Peterek S., Schijlen E.G.W.M., Hall R.D., Bovy A.G., Luo J., Martin C. (2008). Enrichment of tomato fruit with health-promoting anthocyanins by expression of select transcription factors. Nat. Biotechnol..

[bib9] Chouhan S., Sharma K., Zha J., Guleria S., Koffas M.A.G. (2017). Recent advances in the recombinant biosynthesis of polyphenols. Front. Microbiol..

[bib10] Czank C., Cassidy A., Zhang Q., Morrison D.J., Preston T., Kroon P.A., Botting N.P., Kay C.D. (2013). Human metabolism and elimination of the anthocyanin, cyanidin-3-glucoside: a ^13^C-tracer study. Am. Clin. Nutr..

[bib11] Davies K.M., Deroles S.C. (2014). Prospects for the use of plant cell cultures in food biotechnology. Curr. Opin. Biotechnol..

[bib12] Deroles S., Gould K., Davies K.M., Winefield C. (2009). Anthocyanin biosynthesis in plant cell cultures: a potential source of natural colourants. Anthocyanins: Biosynthesis, Functions, and Applications.

[bib13] Downham A., Collins P. (2000). Colouring our foods in the last and next millennium. Int. J. Food Sci. Technol..

[bib14] Dudnik A., Almeida A.F., Andrade R., Avila B., Bañados P., Barbay D., Bassard J.-E., Benkoulouche M., Bott M., Braga A. (2016). BacHBerry: bacterial hosts for production of bioactive phenolics from bERRY fruits. Phytochem. Rev..

[bib15] Espley R.V., Butts C.A., Laing W.A., Martell S., Smith H., McGhie T.K., Zhang J., Paturi G., Hedderley D., Bovy A. (2014). Dietary flavonoids from modified apple reduce inflammation markers and modulate gut microbiota in mice. J. Nutr..

[bib16] Ferrars R., Czank C., Zhang Q., Botting N., Kroon P., Cassidy A., Kay C. (2014). The pharmacokinetics of anthocyanins and their metabolites in humans. Br. J. Pharmacol..

[bib17] Fraser C.M., Thompson M.G., Shirley A.M., Ralph J., Schoenherr J.A., Sinlapadech T., Hall M.C., Chapple C. (2007). Related Arabidopsis serine carboxypeptidase-like sinapoylglucose acyltransferases display distinct but overlapping substrate specificities. Plant Physiol..

[bib18] Giusti M.M., Wrolstad R.E. (2001). Characterization and measurement of anthocyanins by UV‐visible spectroscopy. Curr. Protoc. Food Anal. Chem..

[bib19] Gläßgen W.E., Wray V., Strack D., Metzger J.W., Seitz H.U. (1992). Anthocyanins from cell suspension cultures of *Daucus carota*. Phytochemistry.

[bib20] Goff S.A., Cone K.C., Chandler V.L. (1992). Functional analysis of the transcriptional activator encoded by the maize B gene: evidence for a direct functional interaction between two classes of regulatory proteins. Genes Dev..

[bib21] Heyerdahl P.H., Olsen O.A.S., Hvoslef-Eide A.K., Aitken-Christie J., Kozai T., Smith M.A.L. (1995). Engineering aspects of plant propagation in bioreactors. Automation and Environmental Control in Plant Tissue Culture.

[bib22] Howat S., Park B., Oh I.S., Jin Y.-W., Lee E.-K., Loake G.J. (2014). Paclitaxel: biosynthesis, production and future prospects. New Biotechnol..

[bib23] Hvoslef-Eide A.K., Olsen O.A.S., Lyngved R., Munster C., Heyerdahl P.H. (2005). Bioreactor design for propagation of somatic embryos. Plant Cell, Tissue Organ Cult..

[bib24] Jones J.A., Vernacchio V.R., Collins S.M., Shirke A.N., Xiu Y., Englaender J.A., Cress B.F., McCutcheon C.C., Linhardt R.J., Gross R.A., Koffas M.A.G. (2017). Complete Biosynthesis of Anthocyanins Using E. coli Polycultures. MBio.

[bib25] Kallam K., Appelhagen I., Luo J., Albert N., Zhang H., Deroles S., Hill L., Findlay K., Andersen Ø.M., Davies K. (2017). Aromatic decoration determines the formation of anthocyanic vacuolar inclusions. Curr. Biol..

[bib26] Konczak-Islam I., Okuno S., Yoshimoto M., Yamakawa O. (2003). Composition of phenolics and anthocyanins in a sweet potato cell suspension culture. Biochem. Eng. J..

[bib27] Kovinich N., Kayanja G., Chanoca A., Riedl K., Otegui M.S., Grotewold E. (2014). Not all anthocyanins are born equal: distinct patterns induced by stress in Arabidopsis. Planta.

[bib28] Krisa S., Téguo P.W., Decendit A., Deffieux G., Vercauteren J., Mérillon J.-M. (1999). Production of ^13^C-labelled anthocyanins by *Vitis vinifera* cell suspension cultures. Phytochemistry.

[bib29] Krivoruchko A., Nielsen J. (2015). Production of natural products through metabolic engineering of *Saccharomyces cerevisiae*. Curr. Opin. Biotechnol..

[bib30] Lee E.K., Jin Y.W., Park J.H., Yoo Y.M., Hong S.M., Amir R., Yan Z., Kwon E., Elfick A., Tomlinson S., Halbritter F., Waibel T., Yun B.W., Loake G.J. (2010). Cultured cambial meristematic cells as a source of plant natural products. Nat. Biotechnol..

[bib31] Leonard E., Yan Y., Fowler Z.L., Li Z., Lim C.G., Lim K.H., Koffas M.A. (2008). Strain improvement of recombinant Escherichia coli for efficient production of plant flavonoids. Mol. Pharm..

[bib32] Li D., Wang P., Luo Y., Zhao M., Chen F. (2017). Health benefits of anthocyanins and molecular mechanisms: update from recent decade. Crit. Rev. Food Sci. Nutr..

[bib33] Lim C.G., Wong L., Bhan N., Dvora H., Xu P., Venkiteswaran S., Koffas M.A. (2015). Development of a recombinant escherichia coli strain for overproduction of the plant pigment anthocyanin. Appl. Environ. Microbiol..

[bib34] Luo J., Nishiyama Y., Fuell C., Taguchi G., Elliott K., Hill L., Tanaka Y., Kitayama M., Yamazaki M., Bailey P., Parr A., Michael A.J., Saito K., Martin C. (2007). Convergent evolution in the BAHD family of acyl transferases: identification and characterization of anthocyanin acyl transferases from Arabidopsis thaliana. Plant J..

[bib35] Lyngved R., Snipen L., Iversen T.-H., Hvoslef-Eide A. (2008). Influence of potential growth factors on the production of proembryogenic masses of *Cyclamen persicum* Mill. in bioreactors. Sci. Hortic..

[bib36] Marienhagen J., Bott M. (2013). Metabolic engineering of microorganisms for the synthesis of plant natural products. J. Biotechnol..

[bib37] Miyahara T., Sakiyama R., Ozeki Y., Sasaki N. (2013). Acyl-glucose-dependent glucosyltransferase catalyzes the final step of anthocyanin formation in Arabidopsis. J. Plant Physiol..

[bib38] Mustafa N.R., De Winter W., Van Iren F., Verpoorte R. (2011). Initiation, growth and cryopreservation of plant cell suspension cultures. Nat. Protoc..

[bib39] Nagata T., Nemoto Y., Hasezawa S. (1992). Tobacco BY-2 cell line as the “HeLa” cell in the cell biology of higher plants. Int. Rev. Cytol..

[bib40] Nakamura M., Seki M., Furusaki S. (1998). Enhanced anthocyanin methylation by growth limitation in strawberry suspension culture. Enzyme Microb. Technol..

[bib41] Nielsen I.L.F., Haren G.R., Magnussen E.L., Dragsted L.O., Rasmussen S.E. (2003). Quantification of anthocyanins in commercial black currant juices by simple high-performance liquid chromatography. Investigation of their pH stability and antioxidative potency. J. Agric. Food Chem..

[bib42] Nosov A. (2012). Application of cell technologies for production of plant-derived bioactive substances of plant origin. Appl. Biochem. Microbiol..

[bib43] Nozue M., Kawai J., Yoshitama K. (1987). Selection of a high anthocyanin-producing cell line of sweet potato cell cultures and identification of pigments. J. Plant Physiol..

[bib44] Ochoa-Villarreal M., Howat S., Hong S., Jang M.O., Jin Y.-W., Lee E.-K., Loake G.J. (2016). Plant cell culture strategies for the production of natural products. BMB Rep..

[bib45] Oertel A., Matros A., Hartmann A., Arapitsas P., Dehmer K.J., Martens S., Mock H.-P. (2017). Metabolite profiling of red and blue potatoes revealed cultivar and tissue specific patterns for anthocyanins and other polyphenols. Planta.

[bib46] Oplatowska-Stachowiak M., Elliott C.T. (2017). Food colors: existing and emerging food safety concerns. Crit. Rev. Food Sci. Nutr..

[bib47] Petroni K., Trinei M., Fornari M., Calvenzani V., Marinelli A., Micheli L., Pilu R., Matros A., Mock H.-P., Tonelli C. (2017). Dietary cyanidin 3-glucoside from purple corn ameliorates doxorubicin-induced cardiotoxicity in mice. Nutr., Metab. Cardiovasc. Dis..

[bib48] Ramsay N.A., Glover B.J. (2005). MYB–bHLH–WD40 protein complex and the evolution of cellular diversity. Trends Plant Sci..

[bib49] Roberts S.C. (2007). Production and engineering of terpenoids in plant cell culture. Nat. Chem. Biol..

[bib50] Rodriguez-Amaya D.B. (2016). Natural food pigments and colorants. Curr. Opin. Food Sci..

[bib51] Sasaki N., Nishizaki Y., Ozeki Y., Miyahara T. (2014). The role of acyl-glucose in anthocyanin modifications. Molecules.

[bib52] Scalzo J., Stevenson D., Hedderley D. (2013). Blueberry estimated harvest from seven new cultivars: fruit and anthocyanins. Food Chem..

[bib53] Seitz H.U., Hinderer W., Constabel F., Vasil I.K. (1988). Phytochemicals in plant cell cultures; Anthocyanins.

[bib54] Sigurdson G.T., Tang P., Giusti M.M. (2017). Natural colorants: food colorants from natural sources. Annu. Rev. Food Sci. Technol..

[bib55] Timmers M.A., Grace M.H., Yousef G.G., Lila M.A. (2017). Inter-and intra-seasonal changes in anthocyanin accumulation and global metabolite profiling of six blueberry genotypes. J. Food Compos. Anal..

[bib56] Titta L., Trinei M., Stendardo M., Berniakovich I., Petroni K., Tonelli C., Riso P., Porrini M., Minucci S., Pelicci P. (2010). Blood orange juice inhibits fat accumulation in mice. Int. J. Obes..

[bib57] Tohge T., Nishiyama Y., Hirai M.Y., Yano M., Nakajima J. i., Awazuhara M., Inoue E., Takahashi H., Goodenowe D.B., Kitayama M. (2005). Functional genomics by integrated analysis of metabolome and transcriptome of Arabidopsis plants over‐expressing an MYB transcription factor. Plant J..

[bib58] Tohge T., Zhang Y., Peterek S., Matros A., Rallapalli G., Tandrón Y.A., Butelli E., Kallam K., Hertkorn N., Mock H.P. (2015). Ectopic expression of snapdragon transcription factors facilitates the identification of genes encoding enzymes of anthocyanin decoration in tomato. Plant J..

[bib59] Toufektsian M.C., Lorgeril M., Nagy N., Salen P., Donati M.B., Giordano L., Mock H.P., Peterek S., Matros A., Petroni K., Pilu R., Rotilio D., Tonelli C., de Leiris J., Boucher F., Martin C. (2008). Chronic dietary intake of plant-derived anthocyanins protects the rat heart against ischemia-reperfusion injury. J. Nutr..

[bib60] Wei Y., Ang E.L., Zhao H. (2018). Recent developments in the application of P450-based biocatalysts. Curr. Opin. Chem. Biol..

[bib61] Wilson S.A., Roberts S.C. (2012). Recent advances towards development and commercialization of plant cell culture processes for the synthesis of biomolecules. Plant Biotechnol. J..

[bib62] Yan Y., Li Z., Koffas M.A. (2008). High-yield anthocyanin biosynthesis in engineered Escherichia coli. Biotechnol. Bioeng..

[bib63] Yonekura‐Sakakibara K., Fukushima A., Nakabayashi R., Hanada K., Matsuda F., Sugawara S., Inoue E., Kuromori T., Ito T., Shinozaki K. (2012). Two glycosyltransferases involved in anthocyanin modification delineated by transcriptome independent component analysis in *Arabidopsis thaliana*. Plant J..

[bib64] Yoshida K., Mori M., Kondo T. (2009). Blue flower color development by anthocyanins: from chemical structure to cell physiology. Nat. Product. Rep..

[bib65] Yousuf B., Gul K., Wani A.A., Singh P. (2016). Health benefits of anthocyanins and their encapsulation for potential use in food systems: a review. Crit. Rev. Food Sci. Nutr..

[bib66] Zhang Q., Botting N.P., Kay C. (2011). A gram scale synthesis of a multi-^13^C-labelled anthocyanin,[6, 8, 10, 3′, 5′−13C 5] cyanidin-3-glucoside, for use in oral tracer studies in humans. Chem. Commun..

[bib67] Zhang Y., Butelli E., Alseekh S., Tohge T., Rallapalli G., Luo J., Kawar P.G., Hill L., Santino A., Fernie A.R., Martin C. (2015). Multi-level engineering facilitates the production of phenylpropanoid compounds in tomato. Nat. Commun..

[bib68] Zhang Y., Butelli E., Martin C. (2014). Engineering anthocyanin biosynthesis in plants. Curr. Opin. Plant Biol..

[bib69] Zhou L.-L., Shi M.-Z., Xie D.-Y. (2012). Regulation of anthocyanin biosynthesis by nitrogen in TTG1–GL3/TT8–PAP1-programmed red cells of *Arabidopsis thaliana*. Planta.

