# Supplementary material for: Colour bio-factories: Towards scale-up production of anthocyanins in plant cell cultures
Source: Metab Eng. 2018 Jul;48:218–32. doi: 10.1016/j.ymben.2018.06.004 (PMC6075943; doi:10.1016/j.ymben.2018.06.004)
Supplement: Supplementary file 1 — Supplementary material [file mmc1.pdf]

Supplementary Material

Colour bio-factories: scale-up production of anthocyanins in plant cell cultures

Ingo Appelhagen, Anders Keim Wulff-Vester, Micael Wendell, Anne-Kathrine Hvoslef-Eide, Julia Russell, Anne Oertel, Stefan Martens, Hans-Peter Mock, Cathie Martin, Andrea Matros

Supplementary Methods

1. Generation of suspension cultures

Cell cultures were generated as described in (Mustafa et al., 2011) with the following modifications. Callus formation was induced from darkly pigmented young leaves for approximately 21-28 days on MS agar plates with high auxin to low cytokinin ratio (induction medium, the composition of all media is given in Supplementary Table 1). Explants with developing callus were sub-cultured 2-3 times for 14 days on the same plates. Friable callus was removed from the explants and subsequently transferred to MS plates with reduced phytohormone concentrations (transition medium) and sub-cultured 4-6 times for 14 days. Thereafter, soft callus was kept and continuously sub-cultured for 14 days on MS agar without cytokinins and elevated myo-Inositol concentration (tobacco growth medium). In some cases, callus was transferred directly from induction plates to the final medium, dependent on the formation of soft callus. Cell suspensions were initiated from friable callus and grown as batch cultures at 23°C in the dark at 95 rpm in a rotary shaker (New BrunswickTM Innova® 2000), and were sub-cultured every 7 days.

2. LC-UV/MS and LC-MS/MS analysis of anthocyanins

For LC-UV/MS analysis, 100 µL of the extract were mixed with 25 µL of solvent A (water + 0.5% formic acid) and incubated for one hour at 4°C prior to injection to allow for formation of any potential precipitates. After centrifugation, 5µL of the supernatant were injected per sample. Anthocyanins were analysed by LC-UV/MS as described in Oertel et al. (2017), using an ultra-performance liquid chromatography (UPLC) instrument (H-CLASS, Waters, Milford,

MA, USA) with photo diode array (PDA) detection coupled to an ultra-high resolution time of flight mass spectrometer (UHR-TOF-MS, maXis Impact, Bruker Daltonics, Bremen, Germany) for MS detection. Separation was performed on a CSH Phenyl-Hexyl column (2.1x100mm, 1.7 µm, Waters) equipped with an CSH Phenyl-Hexyl VanGuard pre-column (130 Å, 2.1 x 5 mm, 1.7 µm, Waters) using a gradient from 2% solvent B (acetonitrile + 0.5% formic acid) to 20% solvent B over 4 minutes, followed by a cleaning step from 20% to 98% solvent B over 3 minutes. Column temperature was 35°C and solvent flow rate was 500 µL min^{-1}. UV spectra were acquired from 210 nm to 800 nm. MS were detected after electrospray ionization (ESI) in positive ion mode at 220°C dry temperature, four bar nebulizer gas pressure, 4000 V capillary voltage and a dry gas flow of 11 L min^{-1}. The instrument was operated in MS full scan mode at 3 Hz acquisition speed using the following settings: mass range m/z 50-1500, hexapole radio frequency (RF) voltage 100 V peak-to-peak (Vpp), collision energy 8 V, funnel 1 RF 300 Vpp, funnel 2 RF 600 Vpp, prepulse storage time 15 µs, transfer time 50 µs and collision cell RF 500 Vpp. Instrument calibration was performed with 10 mM sodium formate solution (12.5 mL H_2O, 12.5 mL isopropanol, 50 µL concentrated formic acid, and 250 µL 1M NaOH).

For MS/MS fragmentation analyses the instrument was operated in MS full scan mode and detected by collision-induced dissociation (CID) with argon at 1.5 mTorr in the quadrupole collision cell of the instrument using the following settings: molecular mass 300 Da (width 5 Da) with 27, 22, and 17 eV for charge state 1, 2, and 3 respectively; molecular mass 500 Da (width 6 Da) with 32, 27, and 22 eV for charge state 1, 2, and 3 respectively; molecular mass 1000 Da (width 8 Da) with 45, 40, and 35 eV for charge state 1, 2, and 3 respectively; molecular mass 1500 Da (width 10 Da) with 45, 40, and 35 eV for charge state 1, 2, and 3 respectively.

3. Preparative isolation of anthocyanins from tobacco suspension cultures

Freeze dried cell suspension cultures were ground finely and sequentially extracted with 80% methanol, 2% formic acid (FA) to a final ratio of 100 mL solvent g^{-1} cell dry weight (DW). Insoluble material was removed by centrifugation for 30 min at 13,000 x g. The resulting supernatant was sequentially filtered through 100 µm and 20 µm nylon net filters. The

volume of the filtered extract was reduced under vacuum using a rotary evaporator. Degreasing was undertaken by phase separation with two volumes (v/v) of n-heptane. Any remaining organic solvent was removed under vacuum using a rotary evaporator. Prior to chromatography, the degreased extract was sequentially filtered through nylon net filters down to 0.2 µm. Filtered extract was loaded on a solid phase extraction column (Redi*Sep* RF C18 column, 150 g, 20-40 micron, Teledyne Isco, Lincoln, NE, USA) equilibrated with 2% methanol, 0.5% FA. Anthocyanins were eluted using a linear gradient from 2%-40% methanol, 0.5% FA within 15 column volumes at a flow rate of 30 mL min^{-1} utilizing a Combi*Flash* RF^{+} chromatography instrument (Teledyne Isco) monitored at 280 nm and 515 nm. Fractions showing absorption at 515 nm, were analysed by LC-UV/MS. Fractions containing similar anthocyanins were then combined. The volume and the content of organic solvent of the combined fractions were reduced under vacuum by using a rotary evaporator. For the final purification the fractions were loaded on a preparative HPLC column (CSH Phenyl-Hexyl OBD Prep Column, 130Å, 5 µm, 50 mm X 250 mm, Waters) equilibrated with 2% methanol, 0.5% FA and eluted by a linear gradient from 2%-40% methanol, 0.5% FA within 200 minutes at a flow rate of 20 mL min^{-1} utilising a Varian *ProStar* chromatography instrument (Agilent Technologies, Santa Clara, CA, USA) monitored at 280 nm and 515 nm. The resulting fractions, showing absorption at 515 nm, were analysed by LC-UV/MS and combined. The volume and the content of the organic solvent of the final pure fraction were reduced under vacuum using a rotary evaporator. This fraction was frozen and lyophilised. The dry anthocyanin powder was covered with argon and stored at -20°C until further use.

Literature

Mustafa, N. R., De Winter, W., Van Iren, F., Verpoorte, R., 2011. Initiation, growth and cryopreservation of plant cell suspension cultures. Nature protocols. 6**,** 715.

Oertel, A., Matros, A., Hartmann, A., Arapitsas, P., Dehmer, K. J., Martens, S., Mock, H.-P., 2017. Metabolite profiling of red and blue potatoes revealed cultivar and tissue specific patterns for anthocyanins and other polyphenols. Planta. 246**,** 281-297.

Supplementary Table 1 Growth media. All media were supplemented with plant hormones, 3% (*w/v*) sucrose and vitamins. The modified MS vitamins are based on the original composition, but with increased concentrations of *myo*-Inositol (200 mg L^{-1}) and Thiamine HCl (1 mg L^{-1}). Abbreviations: Murashige & Skoog medium (MS), Gamborg B5 medium (B5), Dichlorophenoxyacetic acid (2,4-D), Naphthaleneacetic acid (NAA), 6-Benzylaminopurine (BAP).

	Callus induction medium	Transition medium	Tobacco growth medium	Arabidopsis growth medium
Medium type	MS	MS	MS	B5
Vitamin mixture	MS	MS	Modified MS	B5
2.4-D (mg L^{-1})	1	1	0.2	0.5
NAA (mg L^{-1})	0.5	-	-	0.5
Kinetin (mg L^{-1})	0.5	0.05	-	0.05
BAP (mg L^{-1})	-	-	-	0.05
Kanamycin (mg L^{-1})	100	100	100	100

Supplementary Table 2 Isotope pattern. Comparison of isotope pattern of C3R from control and ^{13}C-sucrose-labelled cell cultures. For evaluation of the carbon status we extracted the sum of mass spectra across the chromatographic peak for C3R at a retention time of 3.8 minutes for both samples (control and ^{13}C-sucrose). The resulting mass spectra were then compared regarding the isotopic pattern of C3R (chemical formula of the cation $C_{27}H_{31}O_{15}^{+}$). The specified carbon state neglects the incorporation of naturally low abundance ^{2}H (0.0115% relative abundance) and ^{3}H (<0% relative abundance), as well as ^{17}O (0.038% relative abundance) and ^{18}O (0.205% relative abundance). The relative natural abundancies for carbon are 98.93% (^{12}C), 1.07% (^{13}C) and <0% (^{14}C).

**Detected *m/z***	**Ion counts**	**% of monoisotopic mass**	**Carbon state**
Cell culture from AmDel/AmRos1 line with 3% sucrose, control			
**595.1677**	1,330,293	100	27 (12C)
**596.1700**	1,096,041	82.39	26 (12C), 1 (13C)
**597.1723**	211,007	15.86	25 (12C), 2 (13C)
**598.1758**	26,819	2.02	24 (12C), 3 (13C)
**599.1757**	4,157	0.31	23 (12C), 4 (13C)
**600.1846**	872	0.07	22 (12C), 5 (13C)
	2,669,189		

Cell culture from AmDel/AmRos1 line with 3% sucrose with 1/5 ^{13}C-sucrose, ^{13}C-sucrose			
**595.1665**	763,707	100	27 (12C)
**596.1679**	482,184	63.14	26 (12C), 1 (13C)
**597.1730**	544,515	71.30	25 (12C), 2 (13C)
**598.1763**	**565,024**	**73.98**	**24 (12C), 3 (13C)**
**599.1795**	491,082	64.30	23 (12C), 4 (13C)
**600.1829**	409,331	53.60	22 (12C), 5 (13C)
**601.1862**	346,198	45.33	21 (12C), 6 (13C)
**602.1896**	240,406	31.48	20 (12C), 7 (13C)
**603.1928**	182,353	23.88	19 (12C), 8 (13C)
**604.1963**	130,468	17.08	18 (12C), 9 (13C)
**605.1994**	83,620	10.95	17 (12C), 10 (13C)
**606.2033**	54,406	7.12	16 (12C), 11 (13C)
**607.2061**	33,350	4.37	15 (12C), 12 (13C)
**608.2098**	19,300	2.53	14 (12C), 13 (13C)
**609.2131**	11,692	1.53	13 (12C), 14 (13C)
**610.2184**	6,751	0.88	12 (12C), 15 (13C)
**611.2113**	3,889	0.51	11 (12C), 16 (13C)
**612.2239**	2,225	0.29	10 (12C), 17 (13C)
**613.2220**	1,309	0.17	9 (12C), 18 (13C)
**614.2282**	386	0.05	8 (12C), 19 (13C)
	4,372,196		

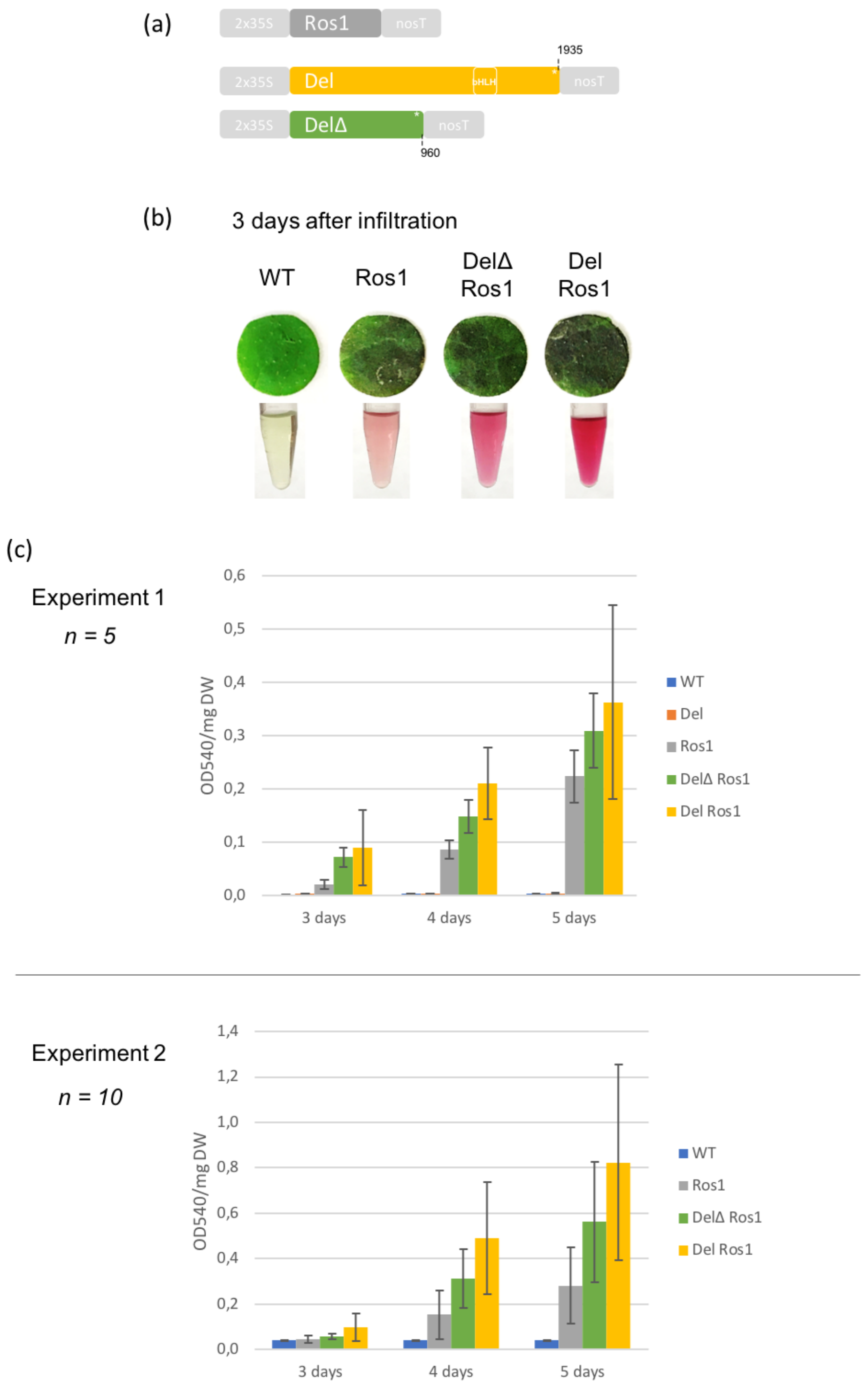

Supplemental Figure 1 Transient expression assays in *N. benthamiana* leaves. (a) The coding sequences of *Am*Ros1, *Am*Del and the truncated version of Delila (*Am*DelΔ) that is encoded by *Am*Del* (*Delila* with premature stop codon) were cloned into the binary vector pMDC32 (Curtis and Grossniklaus, 2003), transformed in *A. tumefaciens* GV3101 pMP90 and co-injected in fully expanded leaves of *N. benthamiana* (Northern Territory accession) plants (OD_{600} 0.2 for each construct). All experiments were done as co-infiltrations with the TBSV anti-silencing suppressor p19. (b) Leaf disks were harvested after 3, 4 and 5 days post infiltration (upper panel shows leaves after 3 days). Anthocyanins were quantified spectrophotometrically in methanol extracts (lower panel). (c) Plants co-infiltrated with *Am*DelΔ and *Am*Ros1 produced more anthocyanins than *Am*Ros1 alone, but less than *Am*Ros1 with the full length *Am*Del.

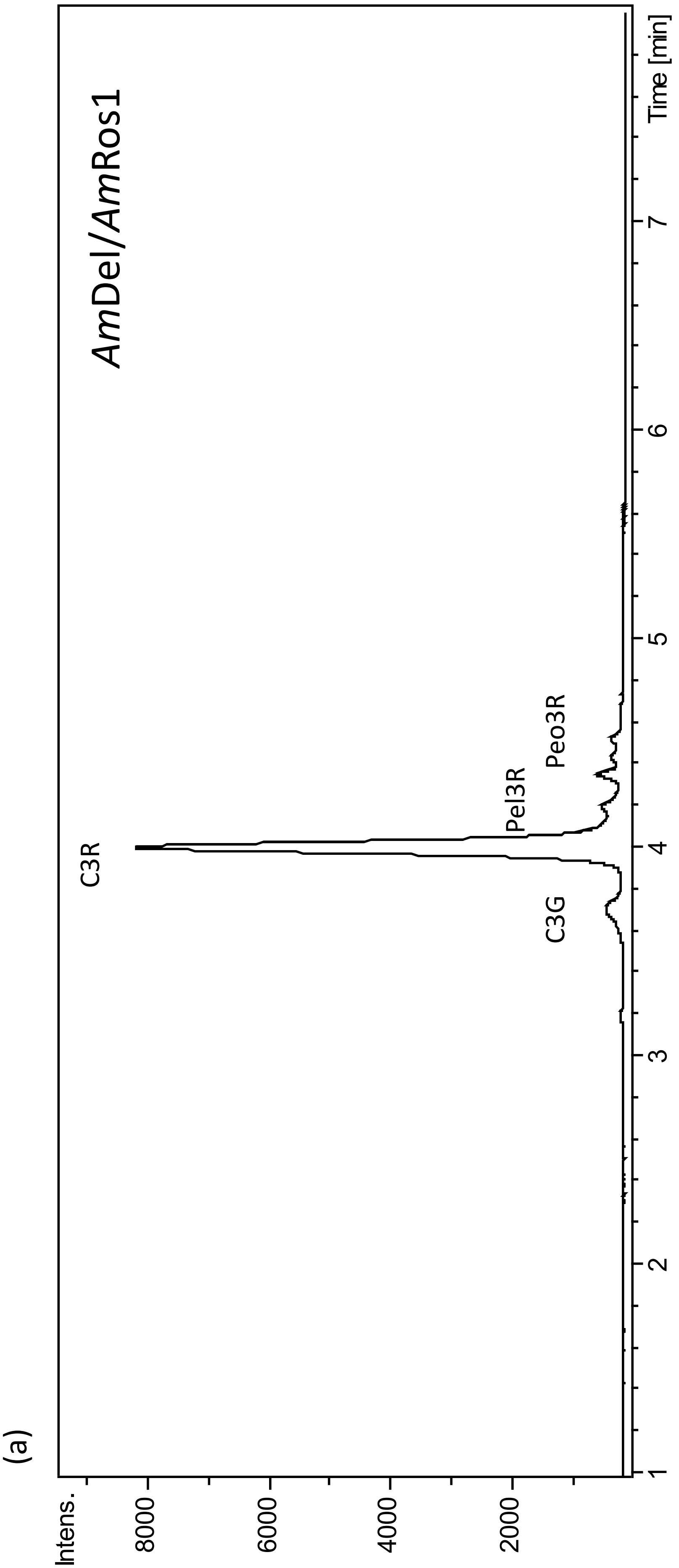
(a)
AmDel/AmRos1
Intens.
8000
6000
4000
2000
C3R
C3G
Pel3R
Peo3R
1
2
3
4
5
6
7
Time [min]

(b)

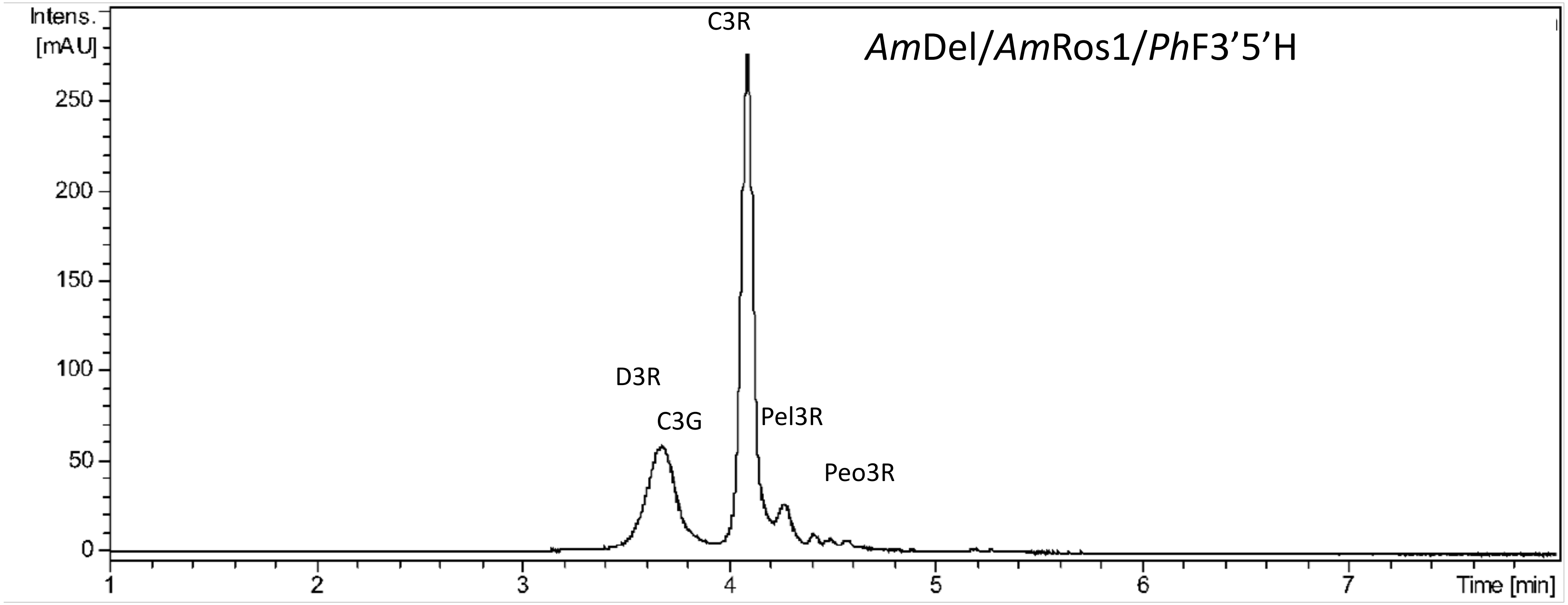
Intens.
[mAU]
250
200
150
100
50
0
1
2
3
4
5
6
7
Time [min]
C3R
AmDel/AmRos1/PhF3'5'H
D3R
C3G
Pel3R
Peo3R

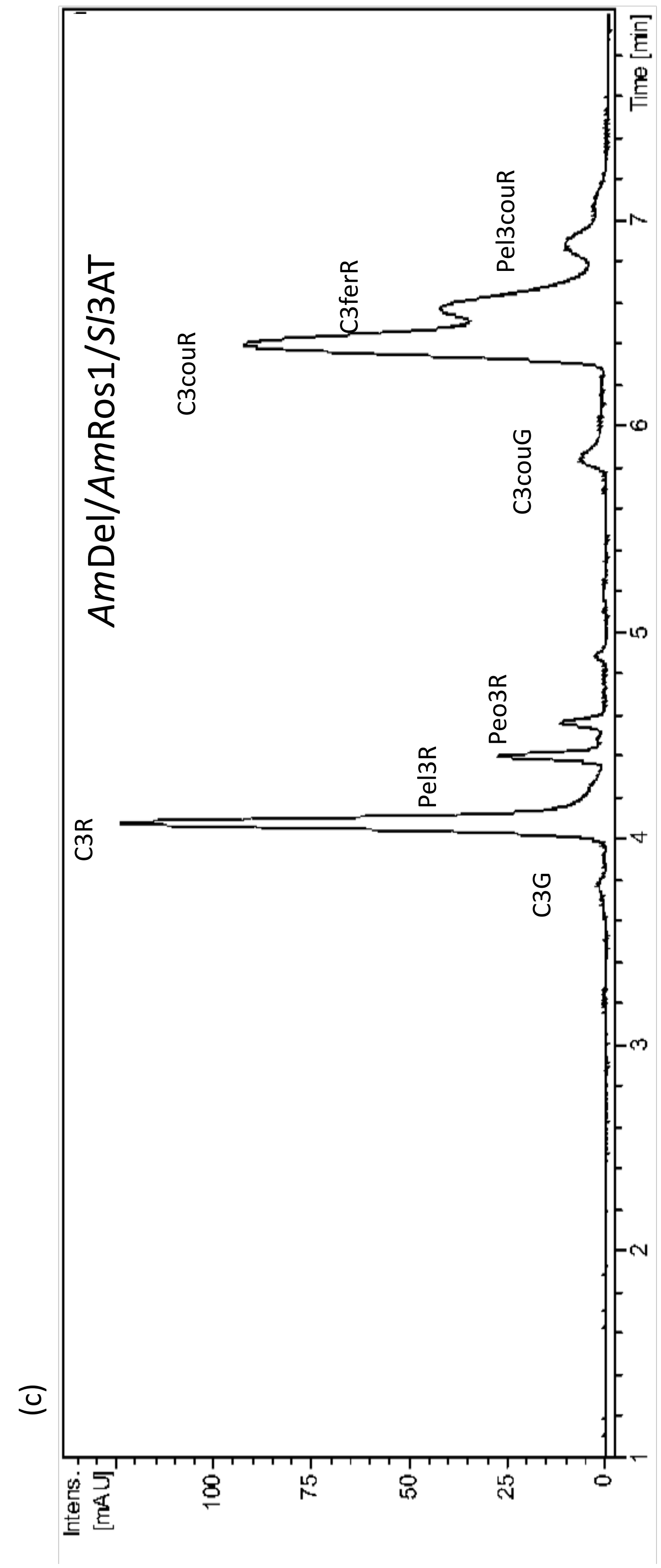

Supplementary Figure 2 Enlarged view of UV-chromatograms as shown in Figure 4. Representative UV-chromatograms at 515 nm for lines expressing (a) AmDel/AmRos1, (b) AmDel/AmRos1/PhF3’5’H and (c) AmDel/AmRos1/Sl3AT. Peak annotation is given in Supplementary Fig. 3.

(a)

Compound	*RetentionTime (min)*	*Molecular Mass, m/z [M]$^+$*	*MS/MS Fragments, m/z [M]$^+$*	*Tentative Structure*
D3R	3.58	611.16	611.16 (D+R)	delphinidin 3-*O*-rutinoside
			465.00 (D+Glc)	delphinidin 3-*O*-glucoside
			303.05 (D)	delphinidin
C3G	3.70	449.11	449.11 (Cy+Glc)	cyanidin 3-*O*-glucoside
			287.05 (Cy)	cyanidin
C3R	3.95	595.17	595.17 (Cy+R	cyanidin 3-*O*-rutinoside
			449.11 (Cy+Glc)	cyanidin 3-*O*-glucoside
			287.05 (Cy)	cyanidin
Pel3R	4.30	579.17	579.17 (Pel+R)	pelargonidin 3-*O*-rutinoside
			433.11 (Pel+Glc)	pelargonidin 3-*O*-glucoside
			271.06 (Pel)	pelargonidin
Peo3R	4.47	609.18	609.18 (Peo+R)	peonidin 3-*O*-rutinoside
			463.12 (Peo+Glc)	peonidin 3-*O*-glucoside
			301.07 (Peo)	peonidin
C3couG	5.78	757.20	757.19 (Cy+cou+Glc)	cyanidin 3-*O* -(coumaroyl) glucoside
			449.11 (Cy+Glc)	cyanidin 3-*O* -glucoside
			287.05 (Cy)	cyanidin
C3couR	6.33	741.40	741.20 (Cy+cou+R)	cyanidin 3-*O* -(coumaroyl) rutinoside
			449.11 (Cy+Glc)	cyanidin 3-*O* -glucoside
			287.05 (Cy)	cyanidin
C3ferR	6.50	771.40	771.21 (Cy+fer+R)	cyanidin 3-*O* -(feruloyl) rutinoside
			449.11 (Cy+Glc)	cyanidin 3-*O* -glucoside
			287.05 (Cy)	cyanidin
Pel3couR	6.83	725.2	725.20 (Pel+cou+R)	pelargonidin 3-*O* -(coumaroyl) rutinoside
			433.11 (Pel+Glc)	pelargonidin 3-*O* -glucoside
			271.06 (Pel)	pelargonidin

(b)

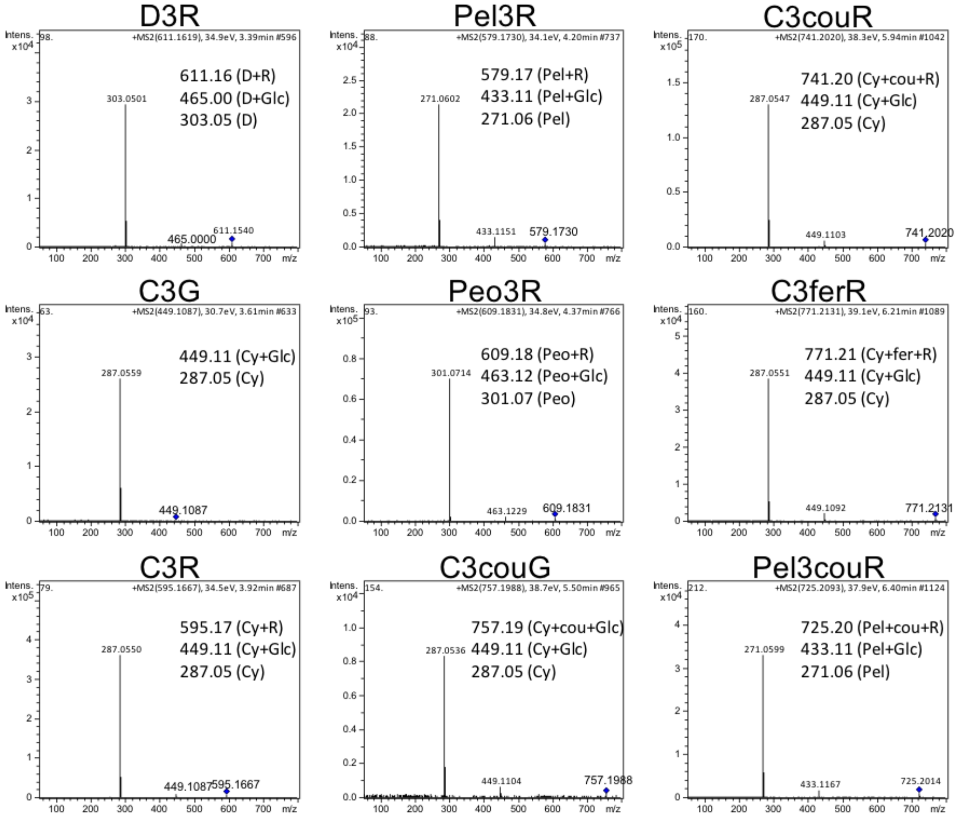

Supplemental Figure 3 (a) Annotation of anthocyanins based on MS fragmentation patterns. (b) Molecular ion mass spectra after collision induced decay are shown for the anthocyanins listed in (a). Abbreviations: delphinidin (D); cyanidin (C); pelargonidin (Pel); peonidin (Peo); petunidin (Pet); glucoside (Glc); rutinoside (R); coumaroyl (cou); feruloyl (fer).

(a)

(b)

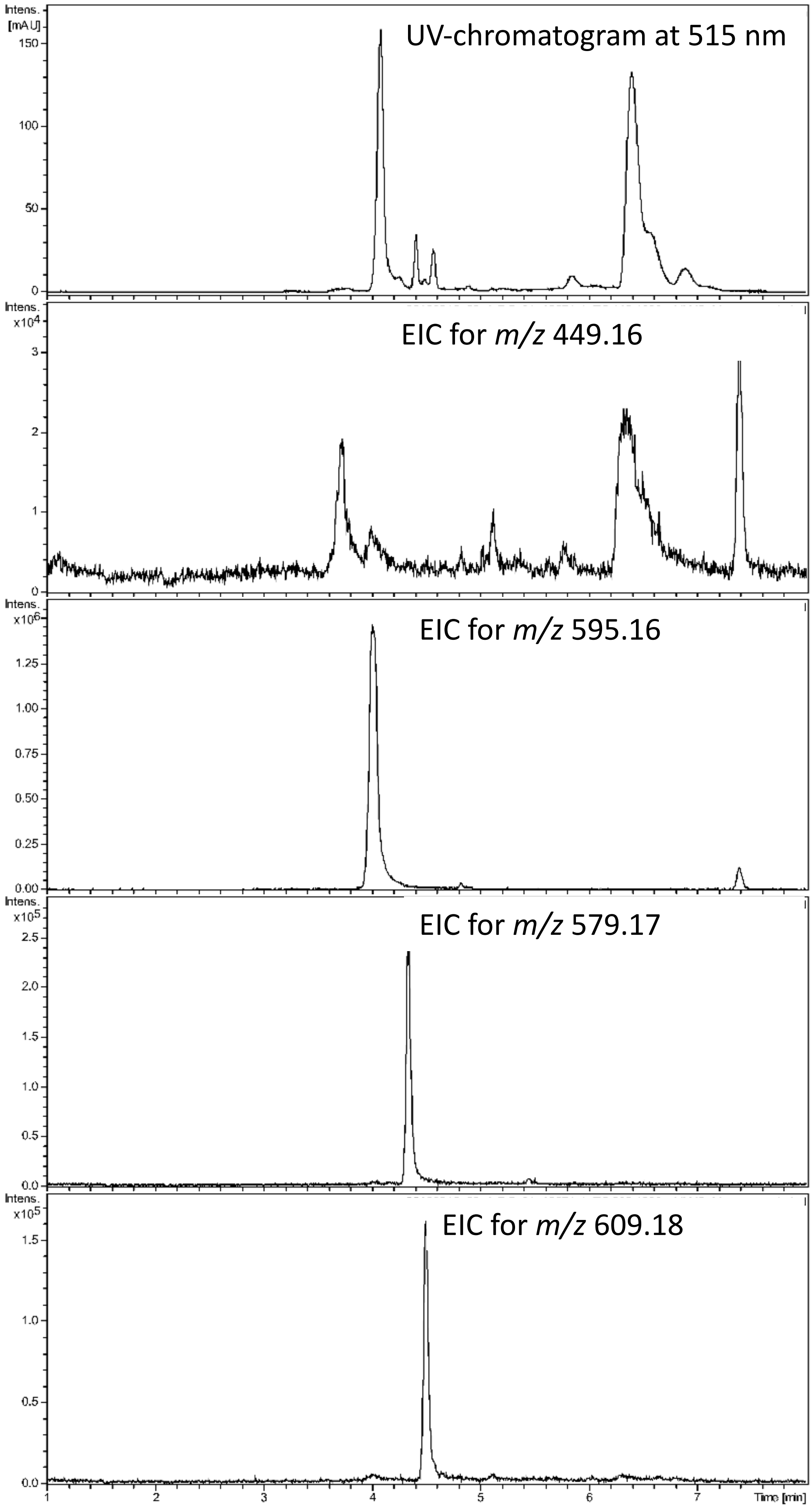

UV-chromatogram at 515 nm
Intens. [mAU]
EIC for m/z 449.16
Intens. x10^4
EIC for m/z 595.16
Intens. x10^6
EIC for m/z 579.17
Intens. x10^5
EIC for m/z 609.18
Intens. x10^5
Time [min]

(b)
continued

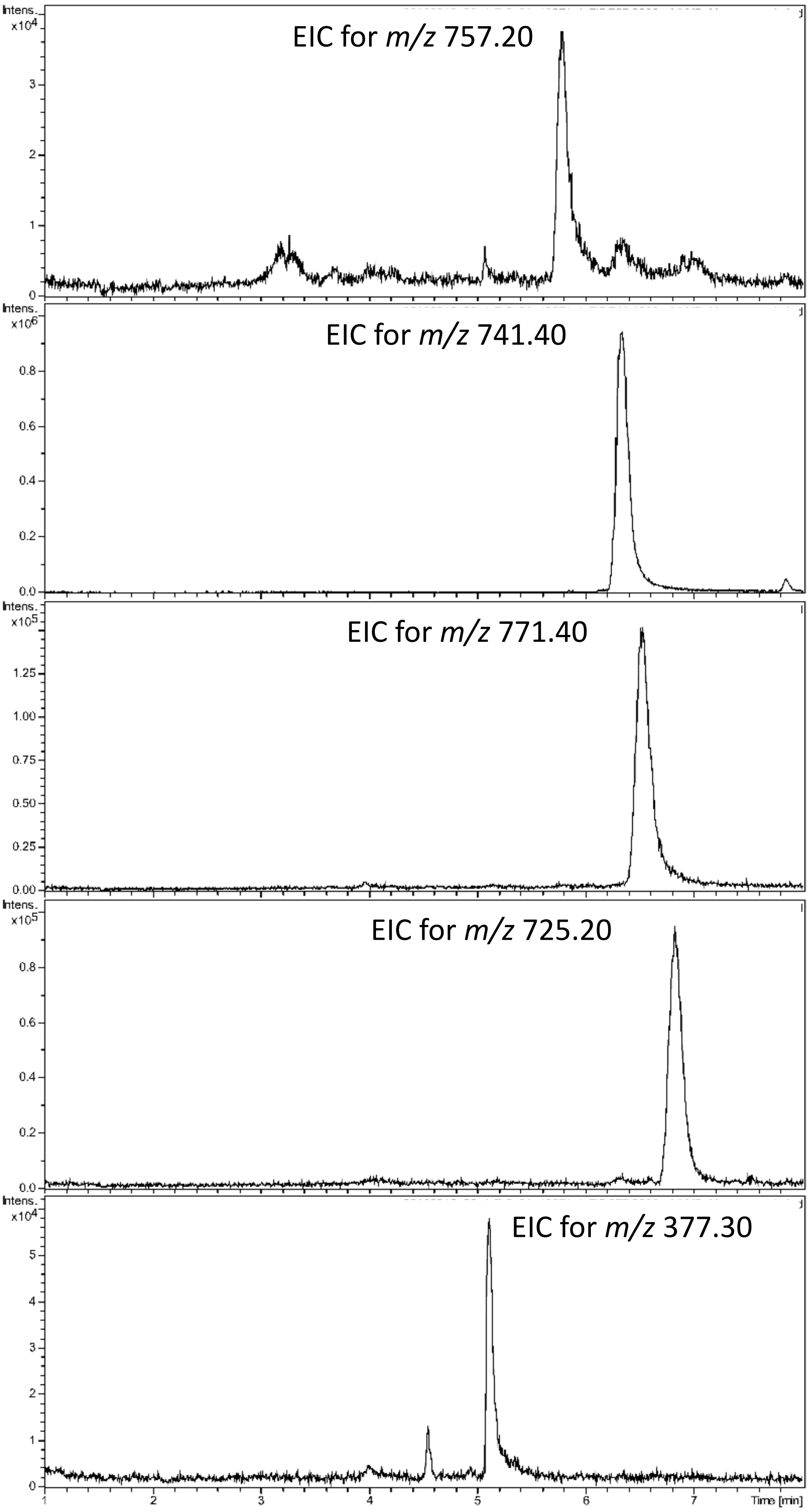

Intens.
x10^4
EIC for m/z 757.20
Intens.
x10^6
EIC for m/z 741.40
Intens.
x10^5
EIC for m/z 771.40
Intens.
x10^5
EIC for m/z 725.20
Intens.
x10^4
EIC for m/z 377.30
Time [min]

(c)

Calculations from integrated peak area from extracted ion chromatograms as presented in Fig. 4 and Supplementary Fig. 5													
	**RT [min]**		**3.58**	**3.70**	**3.95**	**4.30**	**4.47**	**5.78**	**6.33**	**6.50**	**6.83**		**5.14**
	**Molecular mass [M]+**		**611.16**	**449.16**	**595.16**	**579.17**	**609.18**	**757.20**	**741.40**	**771.40**	**725.2**		**377.3 [M+Na]+**
**Batch**	**Line/Molecule**		**D3R**	**C3G**	**C3R**	**Pel3R**	**Peo3R**	**C3couG**	**C3couR**	**C3ferR**	**Pel3couR**	**Total area**	**CGA**
PC25	pBin19		0	0	0	0	0	0	0	0	0		16335
PC32	pBin19		0	0	0	0	0	0	0	0	0		6132
PC60	pBin19		0	0	0	0	0	0	0	0	0		6754
		**mean**	**0**	**0**	**0**	**0**	**0**	**0**	**0**	**0**	**0**		**9740**
		**SD**	**0**	**0**	**0**	**0**	**0**	**0**	**0**	**0**	**0**		**5720**
		**% total area**											
		**% SD**											
PC1	*Am*Del/AmRos1		0	2021471	17897712	2066587	906161	0	0	0	0		271988
PC2	*Am*Del/AmRos1		0	1766277	16317904	2086906	680495	0	0	0	0		288715
		**mean**	**0**	**1893874**	**17107808**	**2076747**	**793328**	**0**	**0**	**0**	**0**	**21871757**	**280352**
		**SD**	**0**	**180449**	**1117093**	**14368**	**159570**	**0**	**0**	**0**	**0**		**11827**
		**% total area**	**0**	**8.7**	**78.2**	**9.5**	**3.6**	**0**	**0**	**0**	**0**		
		**% SD**	**0**	**0.8**	**5.1**	**0.1**	**0.7**	**0**	**0**	**0**	**0**		
PC83A	*Am*Del/AmRos1/ PhF3'5'H		3229101.5	125776	8229806	137566	75292	0	0	0	0		346040
PC83B	*Am*Del/AmRos1/ PhF3'5'H		5331115.5	152395	11998592	153515	100526	0	0	0	0		346896
PC90B	*Am*Del/AmRos1/ PhF3'5'H		4870925.5	147856	11022501	140868	90188	0	0	0	0		322728
PC97B	*Am*Del/AmRos1/ PhF3'5'H		4604710.0	145600	11271857	183002	119925	0	0	0	0		324727
PC102B	*Am*Del/AmRos1/ PhF3'5'H		3787292.0	118015	11198494	184093	111068	0	0	0	0		299953
		**mean**	**4364628.9**	**137928**	**10744250**	**159809**	**99400**	**0**	**0**	**0**	**0**	**15506016**	**328069**
		**SD**	**847173.5**	**15091**	**1454127**	**22476**	**17498**	**0**	**0**	**0**	**0**		**19414**
		**% total area**	**28.2**	**0.9**	**69.3**	**1.0**	**0.6**	**0**	**0**	**0**	**0**		
		**% SD**	**5.5**	**0.1**	**9.4**	**0.1**	**0.1**	**0**	**0**	**0**	**0**		
PC15	*Am*Del/AmRos1/ Sl3AT		0	118987	7027953	605765	629651	310834	7588525	1136513	698127		257330
PC23	*Am*Del/AmRos1/ Sl3AT		0	98279	4909772	509925	380560	216465	4402305	1113266	466459		231818
PC28	*Am*Del/AmRos1/ Sl3AT		0	118936	7867199	640347	451616	277369	7319003	1486095	564456		283724
PC35	*Am*Del/AmRos1/ Sl3AT		0	123395	8310999	788903	506594	287986	7560826	1411631	806916		248036
PC49	*Am*Del/AmRos1/ Sl3AT		0	66889	4133059	622615	215417	121823	2721975	833521	464948		203918
PC56	*Am*Del/AmRos1/ Sl3AT		0	126030	6708302	675381	245438	250514	5074633	1994979	602932		249566
PC70	*Am*Del/AmRos1/ Sl3AT		0	81314	6236251	644633	220058	196822	4236062	2024428	455342		249837
PC91	*Am*Del/AmRos1/ Sl3AT		0	102601	6951969	794821	281909	295670	5622641	1704404	620075		333704
PC98	*Am*Del/AmRos1/ Sl3AT		0	119772	6537264	724689	237149	323516	5953962	1861854	627484		358372
		**mean**	**0**	**111164**	**6520307**	**667453**	**352044**	**253444**	**5608881**	**1507410**	**589638**	**15610341**	**268478**
		**SD**	**0**	**15569**	**1316901**	**90966**	**148850**	**64864**	**1685711**	**422216**	**117742**		**49152**
		**% total area**	**0**	**0.7**	**41.8**	**4.3**	**2.3**	**1.6**	**35.9**	**9.7**	**3.8**		
		**% SD**	**0**	**0.1**	**8.4**	**0.6**	**1.0**	**0.4**	**10.8**	**2.7**	**0.8**		

Supplementary Figure 4 Example of extracted ion chromatograms and integrated peak areas. (a) UV-chromatogram at 515 nm for on cultivated batch of *Am*Del/*Am*Ros1/*Sl*3AT cell culture. (b) Extracted ion chromatograms (EIC) for this batch of *Am*Del/*Am*Ros1/*Sl*3AT cell culture. (c) Integrated peak area as calculated from EIC chromatograms by means of DataAnalysis software (Bruker Daltonics, Bremen, Germany). Abbreviations: delphinidin (D); cyanidin (C); pelargonidin (Pel); peonidin (Peo); petunidin (Pet); glucoside (Glc); rutinoside (R); coumaroyl (cou); feruloyl (fer).

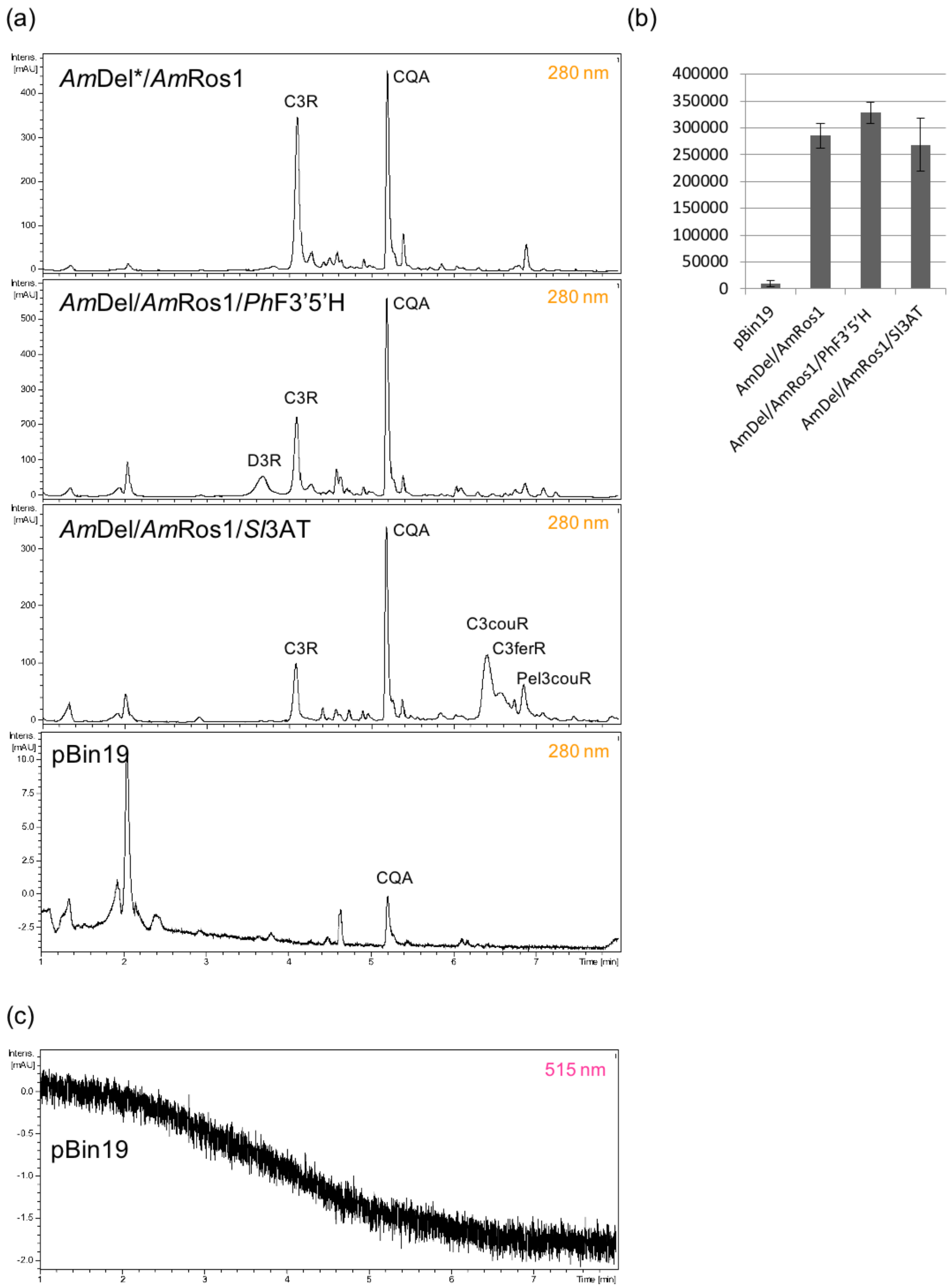

Supplemental Figure 5 Metabolite profiles of different tobacco cell lines. (a) Representative UV-chromatograms at 280 nm. Annotation of peaks refers to Supplementary Fig. 2. (b) Relative amount of caffeoylquinic acid (CQA) in the respective lines; mean integrated peak areas from extracted ion chromatograms are shown. (c) UV-chromatograms of the pBin19 vector control cultures at 515 nm.

(a)

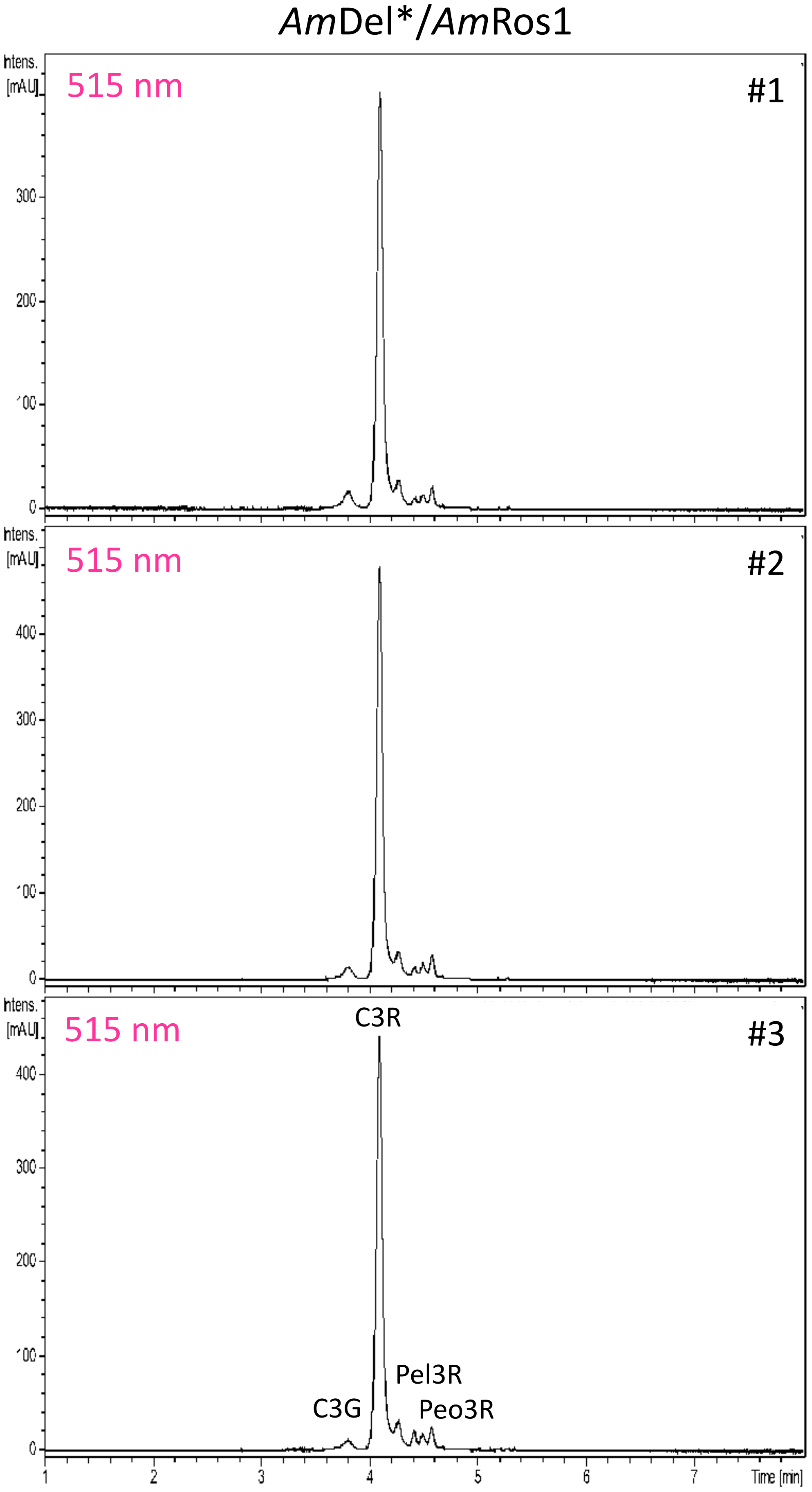
AmDel*/AmRos1
Intens. [mAU]
515 nm
#1
300
200
100
0
515 nm
#2
400
300
200
100
0
515 nm
C3R
#3
400
300
200
100
0
C3G
Pel3R
Peo3R
1
2
3
4
5
6
7
Time [min]

(b)

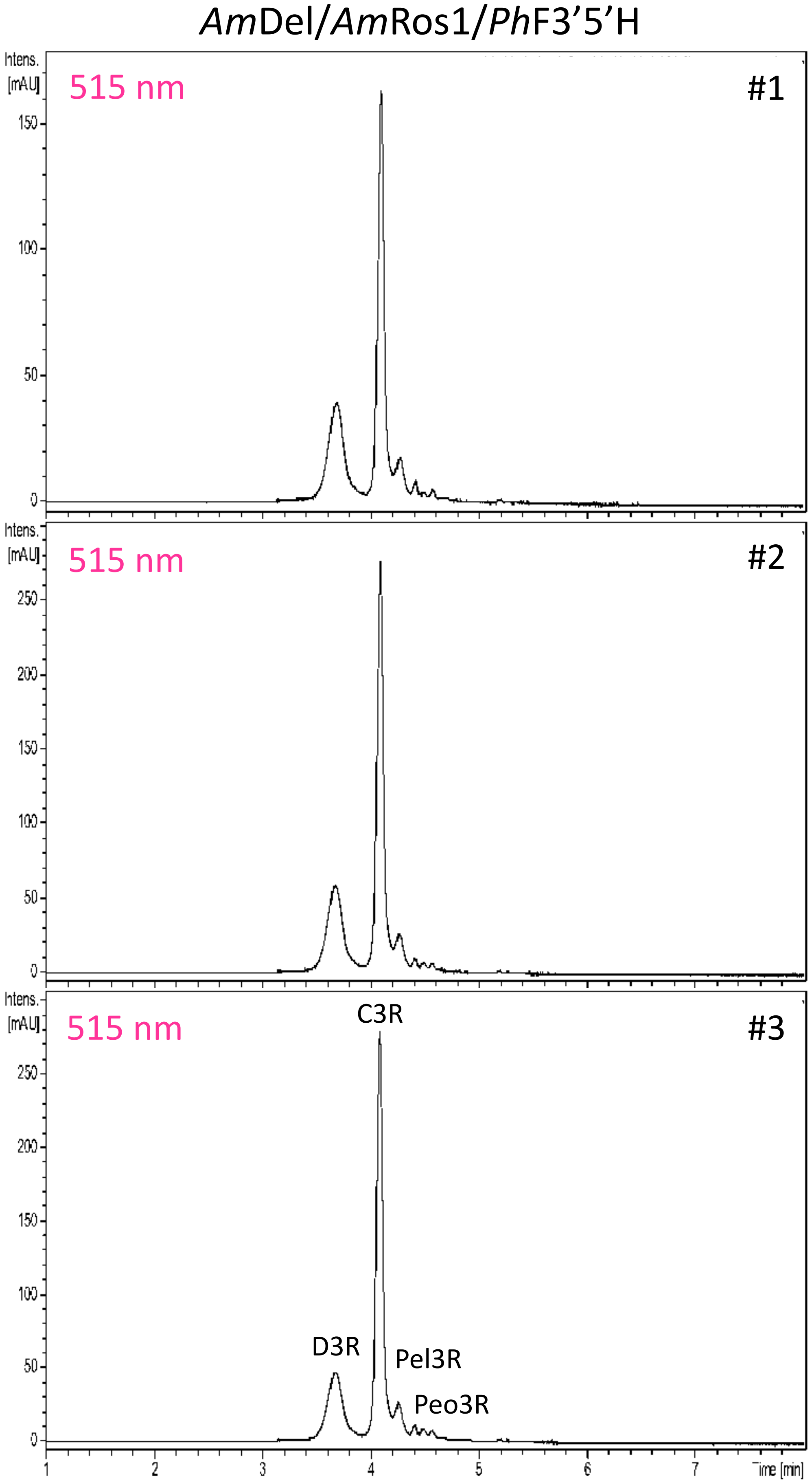
*Am*Del/*Am*Ros1/*Ph*F3'5'H
Intens. [mAU]
515 nm
#1
150
100
50
0
515 nm
#2
250
200
150
100
50
0
515 nm
#3
C3R
D3R
Pel3R
Peo3R
250
200
150
100
50
0
1
2
3
4
5
6
7
Time [min]

(c)

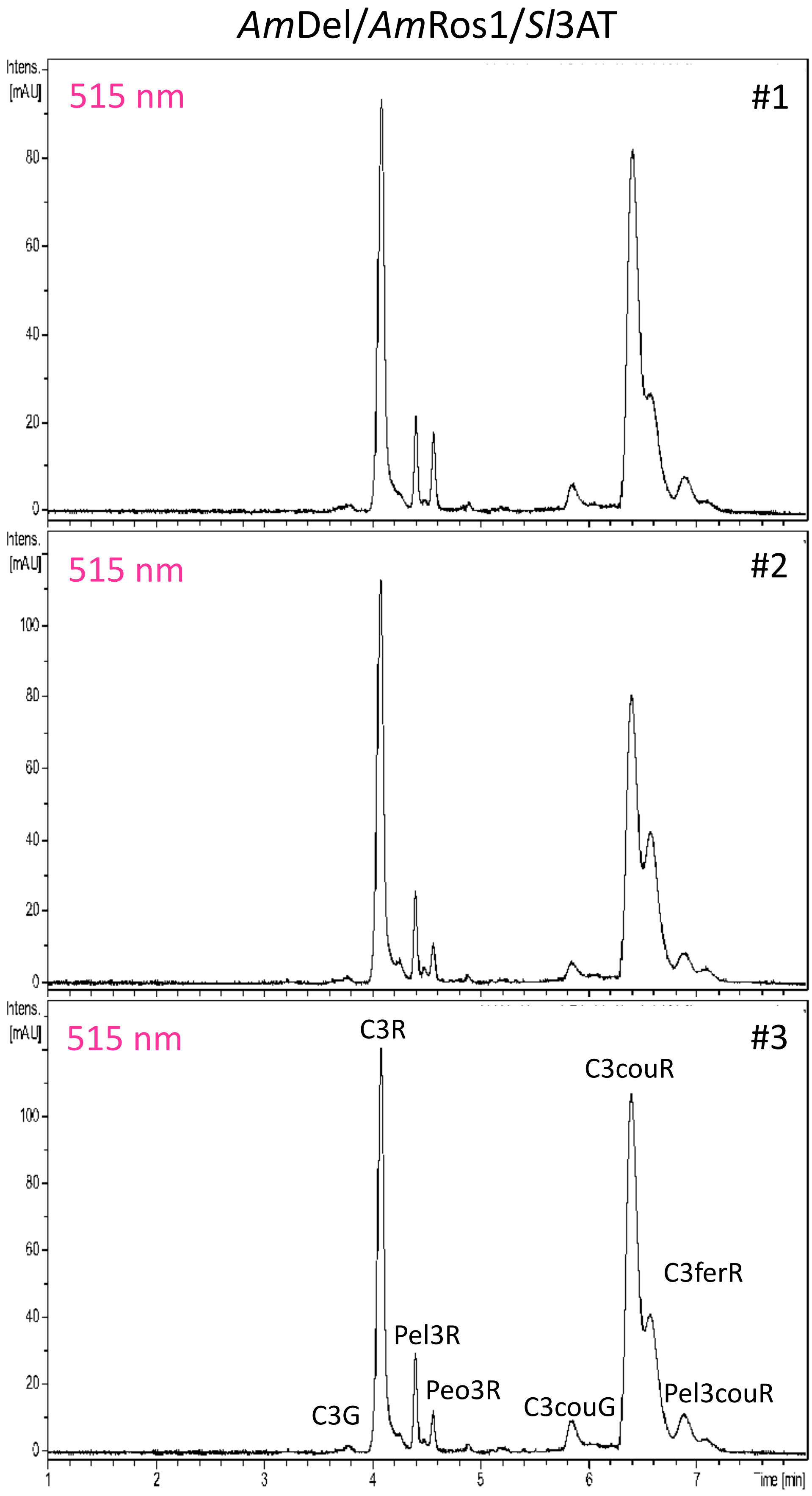
*Am*Del/*Am*Ros1/*Sl*3AT
Intens. [mAU]
515 nm
#1
#2
#3
C3R
C3couR
C3ferR
Pel3R
Peo3R
C3G
C3couG
Pel3couR
Time [min]

(d)

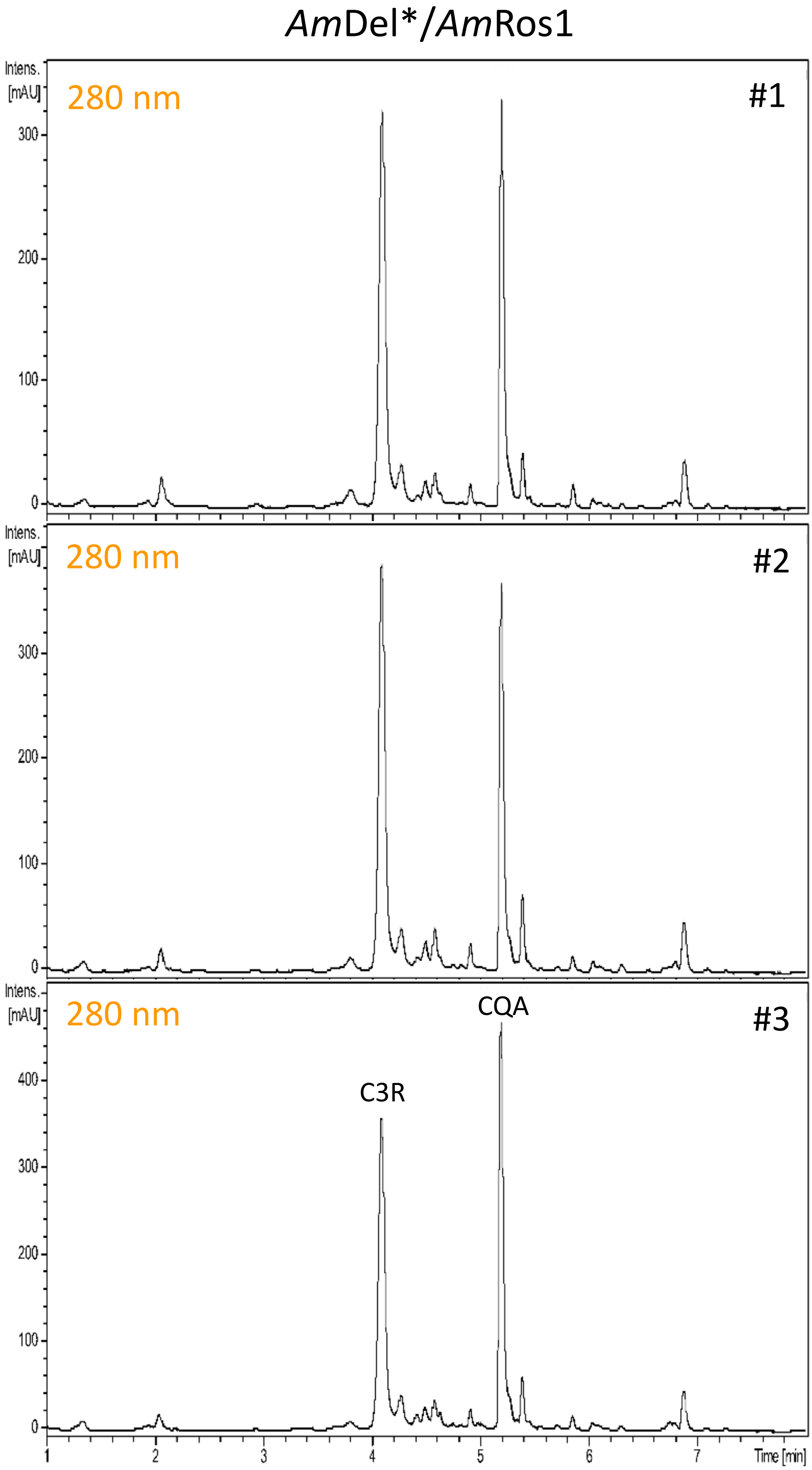
*Am*Del*/*Am*Ros1
Intens. [mAU]
280 nm
#1
300
200
100
0
Intens. [mAU]
280 nm
#2
300
200
100
0
Intens. [mAU]
280 nm
CQA
#3
C3R
400
300
200
100
0
1
2
3
4
5
6
7
Time [min]

*Am*Del/*Am*Ros1/*Ph*F3'5'H

(e)

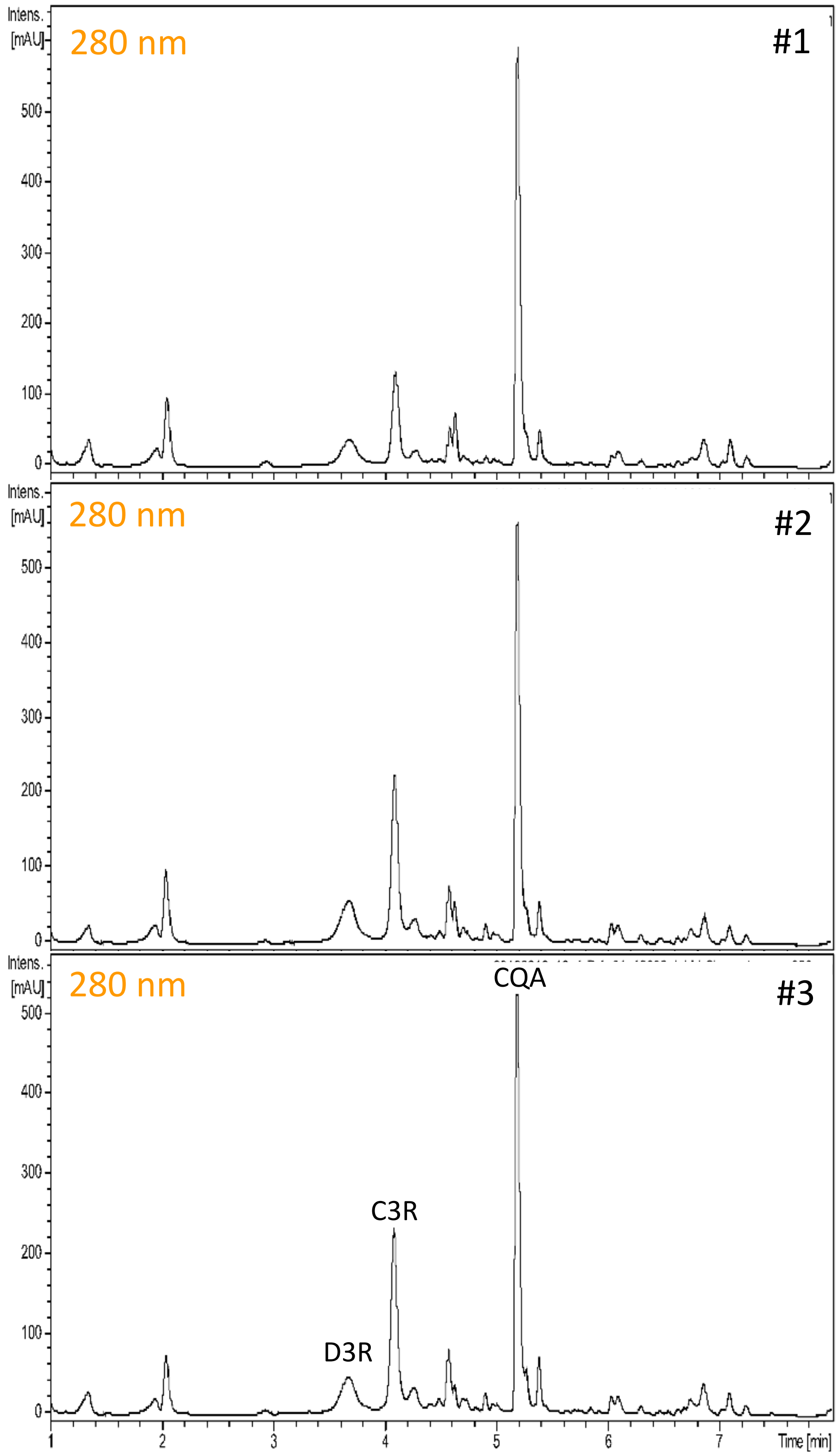

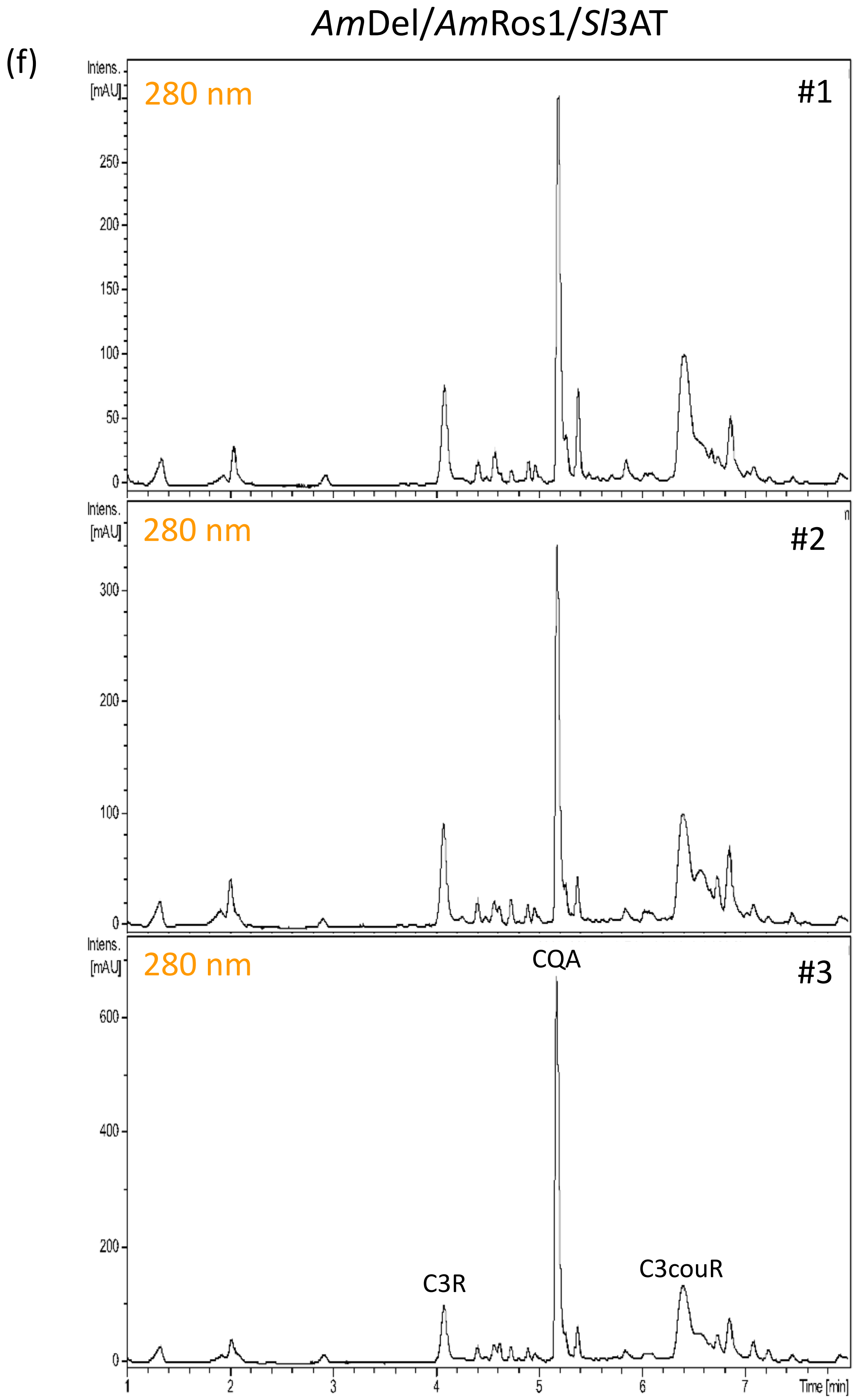

Supplemental Figure 6 Metabolic stability over time. Engineered tobacco cultures were grown over a period of six month and three representative batches (#1, #2 and #3) were analysed by LC-MS. (a) UV chromatograms at 515 nm. (b) UV-chromatograms at 280 nm. For annotation of peaks, refer to Supplementary Fig. 2 and 3.
